# Recent Progress in Reconfigurable and Intelligent Metasurfaces: A Comprehensive Review of Tuning Mechanisms, Hardware Designs, and Applications

**DOI:** 10.1002/advs.202203747

**Published:** 2022-09-18

**Authors:** Yasir Saifullah, Yejun He, Amir Boag, Guo‐Min Yang, Feng Xu

**Affiliations:** ^1^ College of Electronics and Information Engineering Shenzhen University Shenzhen 518060 China; ^2^ School of Electrical Engineering Tel Aviv University Ramat Aviv 69978 Israel; ^3^ Key Laboratory for Information Science of Electromagnetic Waves (MoE) Fudan University Shanghai 200433 China

**Keywords:** intelligent metasurfaces, machine learning, reconfigurable intelligent surfaces, simultaneous transmission and reflection, sixth‐generation, space–time modulation, tunable

## Abstract

Intelligent metasurfaces have gained significant importance in recent years due to their ability to dynamically manipulate electromagnetic (EM) waves. Their multifunctional characteristics, realized by incorporating active elements into the metasurface designs, have huge potential in numerous novel devices and exciting applications. In this article, recent progress in the field of intelligent metasurfaces are reviewed, focusing particularly on tuning mechanisms, hardware designs, and applications. Reconfigurable and programmable metasurfaces, classified as space gradient, time modulated, and space–time modulated metasurfaces, are discussed. Then, reconfigurable intelligent surfaces (RISs) that can alter their wireless environments, and are considered as a promising technology for sixth‐generation communication networks, are explored. Next, the recent progress made in simultaneously transmitting and reflecting reconfigurable intelligent surfaces (STAR‐RISs) that can achieve full‐space EM wave control are summarized. Finally, the perspective on the challenges and future directions of intelligent metasurfaces are presented.

## Graphical abstract

This review presents the recent progress of intelligent metasurfaces, focusing on tuning mechanisms, hardware designs, and applications. The authors discuss the reconfigurable and programmable metasurfaces, classified as space‐gradient, time‐modulated, and space–time modulated metasurfaces. Furthermore, the emerging research direction of reconfigurable intelligent surfaces are demonstrated. Finally, the challenges and future directions of intelligent metasurfaces are presented.

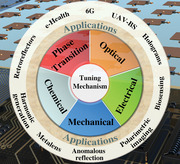

## 1 Introduction

Metamaterials (MTMs), artificially engineered materials composed of subwavelength structures exhibiting unique properties not found in natural materials, have emerged as a new frontier of science involving materials science, engineering, physics, and chemistry. Meta‐atoms are the building blocks that can be arranged into periodic or aperiodic arrays to form 2D metasurfaces and 3D metamaterials, whereas a significantly broader range of material properties can be realized by engineering the geometries and arrangements of the subwavelength building blocks. Modern metamaterials were introduced based on Victor Veselago's theoretical work.^[^ ^1^ ^]^ The first physical metamaterial was developed by John Pendry,^[^ ^2^ ^]^ and David R. Smith first demonstrated a material with negative refractive index experimentally.^[^ ^3^ ^]^ The term “metamaterial” was coined by Walser in 2001.^[^ ^4^ ^]^ In the past two decades, MTMs have been realized in several exciting applications such as electromagnetic cloaks, negative refraction, perfect absorption, and negative permeability and permittivity. While MTMs have introduced several interesting phenomena, several challenges including high loss, fabrication complexity, high fabrication cost, and high weight are prevalent.

Metasurfaces,^[^ ^5^, ^6^ ^]^ the 2D equivalent of metamaterials,^[^ ^7^ ^]^ are artificial structures designed using periodic or aperiodic arrangements of subwavelength meta‐atoms on a typically planar surface. In the last decade, metasurfaces have undergone rapid development and been extensively used to realize numerous novel devices and applications in the microwave,^[^ ^8^ ^]^ terahertz,^[^ ^9^, ^10^ ^]^ and visible regions.^[^ ^11^ ^]^ The refraction and reflection of light at the interface of two homogeneous and isotropic media are governed by Snell's law. In 2011, the generalized Snell's law was proposed,^[^ ^12^ ^]^ and space‐gradient metasurfaces were engineered to undergo abrupt phase change and manipulate EM waves by introducing a phase discontinuity at the interface between two media. Accordingly, metasurfaces have been used to realize a wide range of applications such as holographs,^[^ ^13^, ^14^ ^]^ anomalous reflection,^[^ ^15^, ^16^, ^17^, ^18^ ^]^ orbital angular momentum (OAM),^[^ ^19^, ^20^, ^21^, ^22^, ^23^ ^]^ analog differentiator,^[^ ^24^ ^]^ EM scattering,^[^ ^25^, ^26^, ^27^ ^]^ metalens,^[^ ^28^, ^29^, ^30^, ^31^, ^32^, ^33^, ^34^, ^35^, ^36^, ^37^ ^]^ cloaking,^[^ ^38^, ^39^, ^40^ ^]^ optical encryption,^[^ ^41^ ^]^ quantum information,^[^ ^42^, ^43^ ^]^ and retroreflectors.^[^ ^44^, ^45^ ^]^

Historically, metasurfaces were designed to realize a singular function for a specific incident wave; therefore, they could not realize dynamic functionalities. By designing a multi‐functional metasurface, we can now integrate several functionalities into a single metasurface. Many such designs have been presented to realize polarization‐controlled, wavelength‐selective, and multifunctional metasurfaces. To vary the functionality or operating frequency of passive metasurfaces, redesigning and refabrication are inevitable. Therefore, by tuning the metasurface properties, dynamic control over the incident wave can be realized. In recent years, the tunable, reconfigurable, and programmable metasurfaces concept has been proposed to achieve a higher degree of freedom over electromagnetic waves. Such designs have post‐fabrication reconfigurable characteristics and huge potential in diverse applications. Various tuning mechanisms have been reported in literature that achieve tunability by using materials such as lumped elements,^[^ ^46^, ^47^, ^48^ ^]^ phase‐changing materials,^[^ ^49^, ^50^, ^51^ ^]^ liquid crystals,^[^ ^52^, ^53^, ^54^ ^]^ graphene,^[^ ^55^, ^56^, ^57^, ^58^ ^]^ vanadium‐dioxide,^[^ ^59^ ^]^ and origami‐ and kirigami‐based structures.^[^ ^60^, ^61^, ^62^, ^63^, ^64^, ^65^, ^66^, ^67^, ^68^ ^]^ Further, the principle of a tunable metasurface can be extended to programmable metasurfaces^[^ ^69^, ^70^, ^71^, ^72^, ^73^ ^]^ by embedding a field‐programmable gate array (FPGA) to actively manipulate the EM wave and switch between diverse functions in real time. Such programmable metasurfaces have been extensively implemented in several applications such as imaging,^[^ ^74^, ^75^ ^]^ holograms,^[^ ^41^, ^76^ ^]^ beam focusing,^[^ ^77^, ^78^ ^]^ beam steering,^[^ ^70^, ^79^, ^80^, ^81^, ^82^, ^83^, ^84^, ^85^ ^]^ and wireless communication.^[^ ^86^, ^87^, ^88^, ^89^, ^90^, ^91^, ^92^, ^93^, ^94^, ^95^ ^]^ To realize smart control of EM waves, programmable metasurfaces have been used to develop intelligent metasurfaces with self‐adaptivity, equipped with sensing and feedback components to control their reprogrammable functions without human intervention.^[^ ^96^, ^97^, ^98^, ^99^, ^100^ ^]^ Space‐gradient metasurfaces are constrained by time‐reversal symmetry and Lorentz reciprocity that can be overcome by introducing a temporal gradient metasurface. Hence, spatiotemporal metasurfaces that can realize novel physical phenomena and applications have been proposed,^[^ ^101^, ^102^, ^103^, ^104^, ^105^, ^106^, ^107^, ^108^ ^]^ including breaking the Lorentz reciprocity,^[^ ^109^, ^110^, ^111^, ^112^ ^]^ harmonic manipulations,^[^ ^89^, ^106^, ^113^, ^114^, ^115^, ^116^, ^117^, ^118^, ^119^ ^]^ Doppler cloaks,^[^ ^120^, ^121^, ^122^, ^123^ ^]^ and frequency conversions.^[^ ^124^, ^125^, ^126^, ^127^ ^]^

With recent advancements in metasurfaces, reconfigurable intelligent surfaces (RISs) are being considered as suitable candidates for sixth‐generation (6G) communication.^[^ ^128^, ^129^, ^130^, ^131^, ^132^, ^133^, ^134^ ^]^ Different terminologies have been used to describe RISs in previously published literature, including smart reflector arrays,^[^ ^135^, ^136^ ^]^ intelligent reflecting surfaces (IRS),^[^ ^137^, ^138^, ^139^, ^140^, ^141^, ^142^, ^143^, ^144^ ^]^ large intelligent surfaces,^[^ ^145^, ^146^, ^147^, ^148^, ^149^ ^]^ and passive intelligent mirrors.^[^ ^150^ ^]^ RISs are designed by integrating numerous reconfigurable elements into a 2D structure. They also eliminate the need for transmitter RF chains, making them more economical than conventional multiantenna and relaying technologies. Additionally, RISs are envisioned to be potential candidates for smart radio environments;^[^ ^151^, ^152^, ^153^, ^154^, ^155^ ^]^ they can be applied to several applications such as intelligent transportation systems,^[^ ^156^, ^157^, ^158^ ^]^ Internet of Things (IoT),^[^ ^159^, ^160^ ^]^ unmanned aerial vehicle (UAV)‐based wireless communication,^[^ ^161^, ^162^, ^163^, ^164^, ^165^, ^166^, ^167^ ^]^ and simultaneous wireless information and power transfer (SWIPT) systems.^[^ ^168^, ^169^, ^170^ ^]^ Additionally, simultaneously transmitting and reflecting of reconfigurable intelligent surfaces (STAR‐RISs)^[^ ^171^, ^172^, ^173^ ^]^ have been introduced to overcome the half‐space operation of RISs. Compared to traditional RISs, where the mobile unit could receive only the reflective signal, a STAR‐RIS can receive both reflected and transmitted signals. Consequently, full‐space EM wave control can be achieved using STAR‐RISs.

Several excellent reviews on tunable metasurfaces^[^ ^114^, ^174^, ^175^, ^176^, ^177^, ^178^, ^179^, ^180^ ^]^ and RISs^[^ ^133^, ^151^, ^181^, ^182^ ^]^ have been published. He et al. reviewed the working mechanisms and applications of tunable/reconfigurable metasurfaces.^[^ ^174^ ^]^ A progress report titled “Recent advances in tunable metasurfaces” includes the concept of globally tunable metasurfaces, coding metasurfaces, and software‐defined metasurfaces.^[^ ^175^ ^]^ Zhang et al. presented a comprehensive review of the working principles and applications of space–time coding.^[^ ^114^ ^]^ A survey of smart wireless communications based on IRSs was presented, focusing on performance analysis, diverse applications, and future direction.^[^ ^181^ ^]^ Additionally, Liu et al. reviewed the recent progress in RISs with a focus on operating principles, resource allocation, beamforming, and machine learning‐assisted wireless networks.^[^ ^133^ ^]^

**Figure 1:**
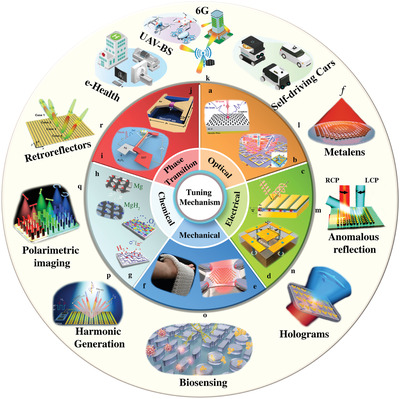
Overview of the diverse functionalities and applications of reconfigurable and intelligent metasurfaces. a) Graphene‐based thin‐film absorber. Reproduced under terms of the CC‐BY license.^[^ ^183^ ^]^ Copyright 2019, the authors, published by American Chemical Society. b) Ultrafast optically tunable metasurface in THz regime. Reproduced with permission.^[^ ^184^ ^]^ Copyright 2019, Wiley‐VCH. c) Electrically tunable metasurface based on ITO. Reproduced with permission.^[^ ^185^ ^]^ Copyright 2016, American Chemical Society. d) Dual‐polarized multifunctional metasurface based on varactor diodes. Reproduced with permission.^[^ ^186^ ^]^ Copyright 2020, Wiley‐VCH. e) Flat optical zoom lens on a stretchable substrate Reproduced with permission.^[^ ^187^ ^]^ Copyright 2016, American Chemical Society. f) Structured fabrics with tunable mechanical properties. Reproduced with permission.^[^ ^188^ ^]^ Copyright 2021, Springer Nature. g) Dynamic color display. Reproduced with permission.^[^ ^189^ ^]^ Copyright 2017, American Chemical Society. h) Dynamic metasurface holograms. Reproduced with permission.^[^ ^190^ ^]^ Copyright 2018, American Association for the Advancement of Science. i) An electrically tunable metasurface based on GST. Reproduced with permission.^[^ ^191^ ^]^ Copyright 2021, Springer Nature. j) Reconfigurable metadevice based on VO_2_. Reproduced with permission.^[^ ^192^ ^]^ Copyright 2017, American Chemical Society. k) Intelligent IoT applications via RISs. Reproduced with permission.^[^ ^159^ ^]^ Copyright 2020, IEEE. l) Reconfigurable varifocal metalens. Reproduced under terms of the CC‐BY 4.0 license.^[^ ^193^ ^]^ Copyright 2021, the authors, published by Springer Nature. m) Optical gap‐surface plasmon metasurfaces for anomalous beam steering. Reproduced under terms of the CC‐BY license.^[^ ^194^ ^]^ Copyright 2020, the authors, published by American Chemical Society. n) Concept of metamaterial hologram. Reproduced under terms of the CC‐BY 4.0 license.^[^ ^195^ ^]^ Copyright 2016, the suthors, published by Springer Nature. o) Dielectric metasurfaces‐based optical biosensors. Reproduced under terms of the CC‐BY license.^[^ ^196^ ^]^ Copyright 2021, the authors, published by American Chemical Society. p) Time‐domain digital coding metasurface‐based harmonic manipulation. Reproduced under terms of the CC‐BY 4.0 license.^[^ ^197^ ^]^ Copyright 2018, the authors, published by Springer Nature. q) Dielectric metasurface for superpixel focusing different polarizations to different spots. Reproduced under terms of the CC‐BY license.^[^ ^198^ ^]^ Copyright 2018, the authors, published by American Chemical Society. r) Reconfigurable spin‐locked metasurface retroreflector. Reproduced under terms of the CC‐BY license.^[^ ^45^ ^]^ Copyright 2022, the authors, published by Wiley‐VCH.

Compared to the aforementioned reviews, this review paper offers a comprehensive review of the tuning mechanisms, practical implementation of intelligent metasurfaces, conventional reflective RISs, and STAR‐RISs (**Figure** **1**). Additionally, we present recent advances in intelligent metasurfaces, covering programmable and space–time digital coding metasurfaces, and prospective research directions for RISs. This paper is organized as follows: In Section 2, we discuss a comprehensive classification of tunable metasurfaces based on tuning mechanisms such as phase transition, optical, electrical, mechanical, and chemical tuning. In Section 3, we discuss digital and programmable metasurfaces, including space‐, time‐, and space–time digital coding metasurfaces. In Section 4, we discuss the fundamental principles of RISs, framework for machine‐learning (ML) empowered RIS systems, and integration of RISs with emerging technologies for 6G wireless networks. In Section 5, an emerging research direction for STAR‐RISs that can manipulate EM waves in full space is introduced. Its performance for different operating protocols, hardware prototypes, and applications is demonstrated. Finally, in Section 6, we conclude this review by presenting our perspective on major research opportunities and emerging research challenges.

## 2 Tunable Metasurface Concepts: Technologies and Approaches

A reconfigurable metasurface can be realized by developing composite or lumped components that can be tuned via external stimuli. Various stimuli, such as voltage source, magnetic polarization field, and ambient temperature, dependent on the nature of the tuning components, can be introduced in the metasurface design. These tuning mechanisms can be broadly classified into global and local tuning. By introducing stimuli‐responsive materials that modify their physical properties according to external ambient stimuli reversibly, entire metasurface functions can be tuned; this approach is referred to as global tuning. Here, the entire metasurface functionality is modified with variations in ambient conditions such as light, electric/magnetic field, pressure, or temperature. Global tuning methods can be classified based on their physical mechanisms such as magnetic, optical, electro‐optical, and thermal mechanisms. Magnetic tuning is based on ferromagnetic resonance (FMR). For optical tuning, light‐sensitive materials, including Si, GaAs, and graphene, have been used. For electro‐optical tuning, graphene and liquid crystals are the two well‐known materials used to design metasurfaces. For thermal tuning, the metasurface is composed of materials that are highly sensitive to temperature variations. The changes in temperature act as a stimulus to control the electromagnetic response of the metasurface. Phase change materials (PCM) are the most attractive solutions for thermally tuning metasurfaces. The phase transition from metal to insulator found in VO_2_ under thermal variation can be utilized to tune the polarization, amplitude, and phase of the scattered fields.

In local tuning, each unit cell is independently tuned. Dynamic functionalities, such as wavefront control and holography, can be achieved by the independent reconfiguration of each unit cell. Local tuning can be achieved by applying most of the global tuning mechanisms at the unit cell level. However, constraints such as crosstalk and modulation speed prohibit some global tuning mechanisms from being used for local tuning. Lumped elements, used for local tuning, can prevent crosstalk. Additionally, they are dynamically reconfigurable and programmable using an FPGA. Most designs presented in previous research have used varactor and PIN diodes to achieve local tuning at the unit cell level. Varactor diodes can be used to realize continuous control over phase and amplitude by varying the voltage applied to the diode. By contrast, PIN diodes can realize binary control over the phase, amplitude, and polarization of EM waves by swapping the diode from the OFF to ON state. The physical mechanism implemented to achieve reconfigurability in metasurface‐based devices is important, as it directly affects the fabrication complexity, cost, scalability, and overall performance of the proposed design.

### 2.1 Tuning via Phase Transitions

Phase‐changing materials (PCM) are considered an effective solution to realize reconfigurable metasurfaces as their refractive index is modified via reversible phase transitions. Employing PCMs in metasurfaces can realize tunable functionalities and their implementation in a multitude of applications. The most commonly used PCMs are germanium–antimony–telluride (Ge_2_Sb_2_Te_5_, also called GST) and VO_2_, which exhibit nonvolatile and volatile switching, respectively.

#### 2.1.1 GST as a PCM

**Figure 2:**
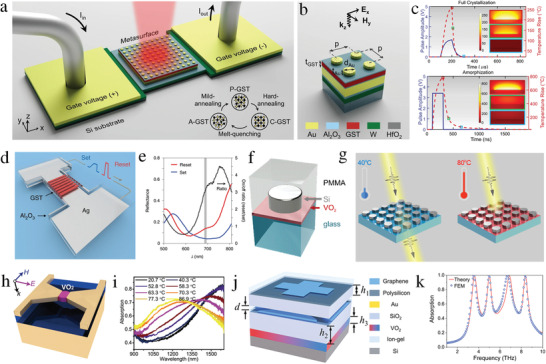
Tuning via phase transitions. a) Electrically driven GST‐based reprogrammable metasurface. b) The schematic of the multi‐layer unit cell. c) The “set” and “reset” pulses for full crystallization and amorphization processes. (a–c) reproduced under terms of the CC‐BY 4.0 license.^[^ ^199^ ^]^ Copyright 2022, the authors, published by Springer Nature. d) The schematic of an electrically tunable metasurface based on GST. e) The binary operation of the perfect absorber metasurface. (d) and (e) reproduced with permission.^[^ ^191^ ^]^ Copyright 2021, Springer Nature. f) A VO_2_‐based tunable dielectric metasurface. g) Tunable functionalities of metasurface realized by temperature variation. (f) and (g) reproduced under terms of the CC‐BY license.^[^ ^200^ ^]^ Copyright 2021, the authors, published by American Chemical Society. h) The schematic diagram of reconfigurable metadevice based on VO_2_. i) Measured results of absorption spectra as a function of the device temperature. (h,i) reproduced with permission.^[^ ^192^ ^]^ Copyright 2017, American Chemical Society. j) The schematic of VO_2_‐based switchable terahertz metasurface unit cell. k) The theoretically calculated absorption spectrum. (j,k) reproduced with permission.^[^ ^201^ ^]^ Copyright 2022, American Chemical Society.

GST is the most common choice as a PCM because it offers fast yet stable phase transition in response to external stimuli and nonvolatile switching. The dielectric characteristics of GST undergo rapid changes during the transition from an amorphous to a crystalline state. For example, an electrically driven GST‐based reconfigurable metasurface with a large tuning range has been developed for optical modulation and wavefront engineering (**Figure** **2**a).^[^ ^199^ ^]^ The developed meta‐switch has an optical efficiency of 80% and spectral tuning over 250 nm. Furthermore, an electrically tunable antenna and metasurface based using GST has also been proposed for spectral tuning, providing more than fourfold electrical modulation of reflectance at 755 nm (Figure 2d).^[^ ^191^ ^]^

#### 2.1.2 Strongly Correlated Materials

VO_2_ is a commonly used PCM as it undergoes an insulator‐to‐metal transition under external electrical, thermal, or optical stimuli. Insulator‐to‐metal transition provides access to a wide range of electrical resistivities and complex refractive indices; hence, VO_2_ has significant potential in tunable metasurfaces. In contrast to the GST phase‐change characteristics, VO_2_ can return to its initial phase without external stimuli. Consequently, VO_2_ undergoes nonvolatile and reversible phase transitions. Further, the phase transition temperature of VO_2_ is lower than that of GST. A reconfigurable metasurface based on Mie‐resonant silicon tunable via a thin layer of VO_2_ has been demonstrated for multifunctional applications (Figure 2f).^[^ ^200^ ^]^ A PCM‐based dynamically reconfigurable metadevice operating in the NIR regime has also been presented, which can significantly reduce the power consumption and switching time owing to strong field concentration in VO_2_ nanocrystals (Figure 2h).^[^ ^192^ ^]^ A VO_2_‐based reconfigurable terahertz metasurface has been applied to quad‐band near‐perfect absorption and antireflection coating (Figure 2j).^[^ ^201^ ^]^ Additionally, a smart optical solar reflector has been developed by integrating the phase transition of VO_2_ with the plasmonic resonance of a metasurface.^[^ ^202^ ^]^ A metal‐to‐insulator phase transition is also observed in VO_2_ under thermal variations. A dual‐polarized programmable metasurface, realized in the THz bands, can be tuned between the “0” and “1” states by varying the biasing voltage applied to VO_2_.^[^ ^203^ ^]^

### 2.2 Optically Tunable Metasurfaces

Optical modulation can be achieved via ultrafast light pulses to realize a tunable metasurface by using light‐sensitive materials in the metasurface. Semiconductors are appropriate for this application as their conduction carriers are dependent on optical pumping. Other materials used to realize optically tunable metasurfaces are graphene and indium tin oxide ITO.

#### 2.2.1 Semiconductors

**Figure 3:**
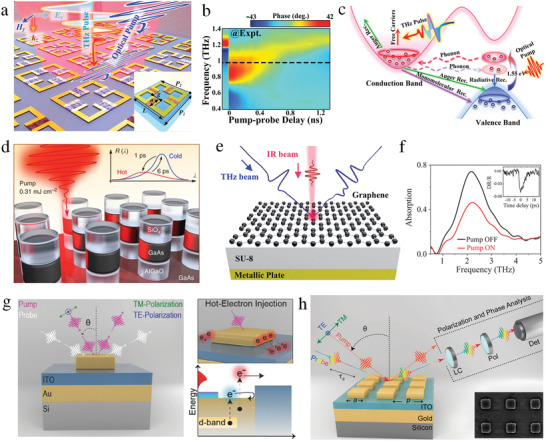
Optically tunable metasurfaces. a) Schematic illustration of ultrafast optically tunable THz metasurface. b) Measured phase spectra as a function of the pump–probe time delay. c) The sketch of photoexcited carrier transitions in the Si bridges. Reproduced with permission.^[^ ^184^ ^]^ Copyright 2019, Wiley‐VCH. d) The conceptual illustration of optical tuning of direct‐gap semiconductor metasurfaces. Reproduced under terms of the CC‐BY 4.0 license.^[^ ^204^ ^]^ Copyright 2017, the authors, published by Springer Nature. e) A graphene‐based thin‐film absorber. f) The carriers are excited by an infrared pump beam. (e) and (f) reproduced with permission.^[^ ^183^ ^]^ Copyright 2019, American Chemical Society. g) Schematic of the all‐optical modulation based on the plasmonic lattice. Reproduced with permission.^[^ ^205^ ^]^ Copyright 2018, Wiley‐VCH. h) All‐optical phase and polarization modulation characterization setup. Reproduced with permission.^[^ ^206^ ^]^ Copyright 2018, American Chemical Society.

Si and GaAs are the most commonly used semiconductors for realizing optically tunable metasurfaces as they can be switched from an insulator to a metal via optically excited carriers. Various tunable metasurface designs have been reported based on split ring resonators (SRRs) operating in the THz band.^[^ ^207^, ^208^, ^209^ ^]^ Conversely, Si can be used in a tunable metasurface to switch from a dielectric to a conductor‐like state based on the photoexcitation of the carriers. Each unit cell of frequency‐tunable metasurface was composed of four SRRs separated by four Si bridges. The optical excitation enables a significant increase in the electron–hole plasma density in Si bridges, leading to wide ranges of resonant frequency tuning and phase shifting. (**Figure** **3**a).^[^ ^184^ ^]^ Additionally, a direct‐gap semiconductor‐based all‐optical tunable metasurface was presented. (Figure 3d).^[^ ^204^ ^]^ Arrays of Mie‐resonance‐based GaAs nanodisks also offer ultrafast and low‐power optical modulation.

#### 2.2.2 Graphene

Graphene, a 2D form of carbon, exhibits several unique optical, electrical, mechanical, and thermal properties. Among many of the unique properties of graphene, its reconfigurable optical properties allow it to be used in many photonic and plasmonic applications. Recently, many graphene‐based optically tunable metasurfaces have been reported.^[^ ^210^, ^211^ ^]^ Further, ultrafast tunable intensity modulation has been achieved via an optical pump signal applied to a graphene‐based ultrathin absorber in the THz band (Figure 3e).^[^ ^183^ ^]^

#### 2.2.3 Transparent Conducting Oxides

Transparent conductive oxide (TCO)‐based devices exhibit optically induced refractive index modifications. ITO and aluminum‐doped zinc oxide (AZO) are a class of wide‐bandgap semiconductors that can be highly doped without altering the material structure. Therefore, ITO a suitable candidate for reconfigurable photonic systems. Furthermore, ultrafast modulation of polarization and phase in the visible region was demonstrated by on‐resonance pumping of the device, facilitating hot‐electron transfer from gold nanoparticles to the ITO layer (Figure 3g).^[^ ^206^ ^]^

### 2.3 Electrically Tunable Metasurfaces

#### 2.3.1 Liquid Crystals

**Figure 4:**
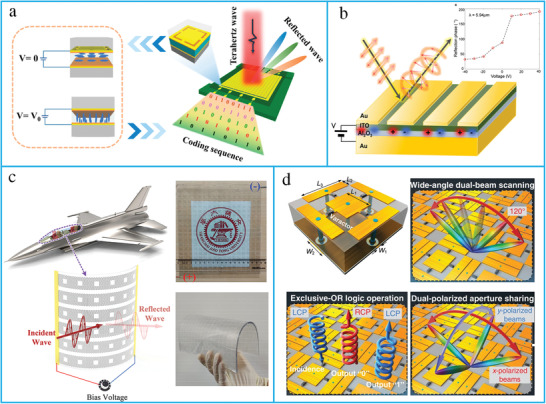
Electrically tunable metasurfaces. a) The conceptual illustration of liquid‐crystal‐integrated programmable metasurfaces array for THz beam steering. Reproduced with permission.^[^ ^82^ ^]^ Copyright 2022, American Chemical Society. b) An electrically tunable metasurface based on ITO. Reproduced with permission.^[^ ^185^ ^]^ Copyright 2016, American Chemical Society. c) The schematic of the graphene‐based optically transparent metasurface. Reproduced with permission.^[^ ^217^ ^]^ Copyright 2021, Elsevier. d) Dual‐polarized multifunctional metasurface based on varactor diodes. Reproduced under terms of the CC‐BY license.^[^ ^186^ ^]^ Copyright 2020, the authors, published by Wiley‐VCH.

Liquid crystals (LCs) are considered promising candidates for realizing tunable metasurfaces as they exhibit distinct phase responses to external stimuli. By varying the electric/magnetic field, pressure, and temperature, LCs can modify the polarization and direction of an incident wave. An LC‐based programmable metasurface was developed for THz beam manipulation (**Figure** **4**a).^[^ ^82^ ^]^ By electrically tuning LC molecules, the dielectric constant changes, and a phase difference of nearly 180° is achieved between the biased and unbiased states. A commercial dynamic metasurface antenna prototype based on an LC was developed for synthetic aperture radar (SAR) imaging in the X‐band.^[^ ^212^ ^]^ Further, nematic liquid crystals (NLCs), based on millimeter‐wave digital coding metasurfaces, have been developed, wherein different reflection phases are achieved by tuning the bias voltage of the NLCs.^[^ ^213^ ^]^ LC‐assisted THz devices have low fabrication complexity, compactness, light weight, and lower cost compared to electrically tunable devices. A programmable metasurface based on an LC that can tune the phase distribution dynamically to realize beam steering at in the THz band has been designed.^[^ ^80^ ^]^ An LC‐assisted programmable transmissive metasurface has also been designed to achieve diverse functionalities such as orbital angular momentum, multiple beams, and dual beam steering.^[^ ^83^ ^]^ LCs are probably the most used tuning mechanism in electro‐optical applications via external electric and magnetic fields. An LC‐based dielectric metasurface comprising of silicon nanodisks embedded into LCs was designed.^[^ ^214^ ^]^ By electrically tuning LCs, a large spectral shift is induced in the metasurface resonance, resulting in a transmission modulation of 75%. Dynamic varifocal meta‐lenses and switchable meta‐holograms are realized in the visible region using an LC‐based multifunctional polarization‐dependent metasurface.^[^ ^11^ ^]^ If the response time and ambient temperatures are not critical issues, LCs are a robust and affordable technology to engineer reconfigurable metasurfaces.

#### 2.3.2 Transparent Conducting Oxides

Transparent conducting oxides (TCOs) have gained considerable attention for application in tunable metasurfaces in the near‐infrared (NIR) and mid‐infrared (MIR) regimes as they can be doped to high carrier concentrations, allowing for infrared (IR) guiding surface modes at the interface of a conducting oxide and air.^[^ ^215^ ^]^ For example, by electrically tuning the carrier densities of ITO inside plasmonic resonator arrays, the reflection phase of light can be dynamically controlled (Figure 4b).^[^ ^185^ ^]^ Furthermore, electrically tunable optical response based on an ionic conductance mechanism with reflectance modulation of up to 78% and low modulation voltage of 100 mV has been demonstrated.^[^ ^216^ ^]^ Devices based on the electrical depletion of TCOs have smaller depletion widths due to the screening effect, thereby limiting modulation depth. The modulation depth can be increased by using multiple gates or an optical pump.

#### 2.3.3 Graphene

Graphene, a monolayer carbon, has attracted significant attention for its application in tunable devices and structures. A graphene‐based optically transparent metasurface has been presented for tunable absorption (Figure 4c).^[^ ^217^ ^]^ By varying the bias voltage supplied to graphene, a tunable absorber can be realized in different bands. Additionally, a tunable metasurface in THz frequencies has been designed for absorption enhancement by incorporating graphene sheets in cut‐wire arrays.^[^ ^218^ ^]^ A gate‐controlled graphene‐based metasurface has also been designed in the THz regime for wideband tunable phase modulation.^[^ ^57^ ^]^ A graphene‐based multi‐bit metasurface has been demonstrated to control THz wavefronts, arbitrary beam manipulation, and orbital angular momentum in real time.^[^ ^21^ ^]^ Graphene, with its unique crystalline and electronic structure, has outstanding optoelectronic properties that can be tuned via external stimuli. The tunable optoelectronic devices can be designed by integrating graphene in the metasurface structures to achieve higher speed, amplitude, and stability. A plasmonic modulator has been designed based on a groove‐structured metasurface and single layer of graphene, which can achieve ten times higher modulation depth as compared with conventional plasmonic modulators.^[^ ^219^ ^]^

#### 2.3.4 Tuning via Lumped Element

The most popular tuning mechanism for reconfigurable intelligent surfaces in microwave frequencies is lumped elements. PIN diodes and varactors are most commonly used, and DC biasing is applied to tune their impedance and control reconfigurable functions. Accordingly, a programmable metasurface based on electrically tuned varactors has been developed to can manipulate *x*‐ and *y*‐polarized waves independently (Figure 4d).^[^ ^186^ ^]^ Additionally, tunable spatial phase‐shifting of a programmable metasurface can be realized using PIN diodes. Metasurface‐based smart wireless power transfer systems can realize dynamic indoor charging based on a near‐field focusing technique.^[^ ^220^ ^]^ Furthermore, a binary programmable metasurface has been proposed to synthesize sum and difference beams useful for detecting and tracking targets in monopulse radar systems.^[^ ^221^ ^]^ A varactor diode‐based tunable meta‐atom has also been proposed to realize a 3‐bit angle‐insensitive RIS with phase coverage of 315°.^[^ ^222^ ^]^ However, lumped elements are not suitable for applications at frequencies above the GHz range due to the operating frequencies of PIN diodes and varactors.

### 2.4 Mechanically Tunable Metasurfaces

Mechanical metamaterials are artificial structures designed by the spatial arrangement of 2D and 3D building blocks and meta‐atoms that deform, rotate, buckle, fold, and snap in response to mechanical forces. Several excellent reviews of active mechanical metamaterials have been published.^[^ ^66^, ^223^, ^224^, ^225^, ^226^, ^227^, ^228^, ^229^ ^]^ We have reviewed recent developments in mechanically tunable metasurfaces based on tuning techniques such as microelectromechanical systems (MEMS), microfluidics, mechanically stretchable substrates, and kirigami/origami‐based metasurfaces.

#### 2.4.1 MEMS‐Based Tunable Metasurfaces

**Figure 5:**
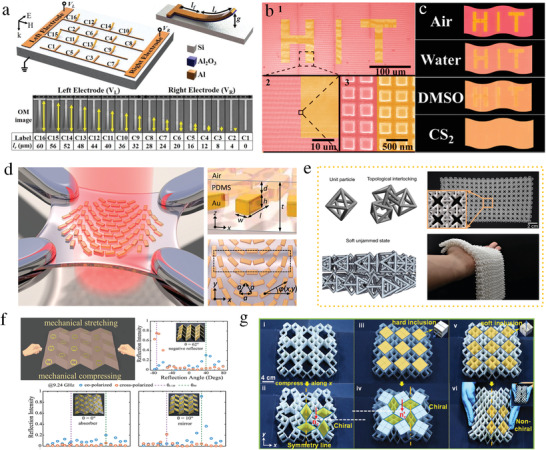
Mechanically tunable metasurfaces. a) Schematics of MEMS‐based tunable metamaterials for THz bandwidth control. Reproduced with permission.^[^ ^240^ ^]^ Copyright 2017, AIP Publishing. b) The SEM images of a microfluidic reconfigurable all‐dielectric metasurface. The information and background are designed by two types of metasurfaces. c) The photograph of samples in different solvents. (b) and (c) reproduced with permission.^[^ ^241^ ^]^ Copyright 2018, American Chemical Society. d) The schematic illustration of the flat optical zoom lens on a stretchable substrate. Reproduced with permission.^[^ ^187^ ^]^ Copyright 2016, American Chemical Society. e) Conceptual illustration of structured fabrics with tunable mechanical properties. Reproduced with permission.^[^ ^188^ ^]^ Copyright 2021, Springer Nature. f) The conceptual illustration of origami metawall which can act as a negative reflector, absorber, and mirror under external mechanical stimuli. Reproduced under terms of the CC‐BY license.^[^ ^34^ ^]^ Copyright 2019, the authors, published by Wiley‐VCH. g) The prototype of 3D transformable kirigami‐based programmable metamaterials. Reproduced with permission.^[^ ^63^ ^]^ Copyright 2021, Wiley‐VCH.

MEMS are suitable candidates for realizing active metamaterials due to their reconfigurable mechanical structures, low power consumption, and compatibility with complementary metal‐oxide‐semiconductor (CMOS) technology. Several reviews of MEMS‐based active mechanical metamaterials have been published.^[^ ^230^, ^231^, ^232^ ^]^ MEMS‐based mechanically tunable metasurfaces are classified by their actuator mechanisms such as piezoelectric,^[^ ^233^ ^]^ electrothermal,^[^ ^234^, ^235^, ^236^ ^]^ and electrostatic actuation.^[^ ^237^, ^238^, ^239^ ^]^ A MEMS‐based broadband tunable metamaterial has been proposed for operation in the THz region using cantilever resonators of varying release lengths (**Figure** **5**a).^[^ ^240^ ^]^

#### 2.4.2 Microfluidics‐Based Tunable Metasurfaces

Microfluidics has been applied to realize active mechanical metamaterials, specifically in biosensing applications. Their optical responses vary reversibly when empty microfluidic channels are filled with liquid metal. A real‐time reconfigurable metasurface has been proposed to realize tunable colors using microfluidics.^[^ ^241^ ^]^ By mechanically injecting and ejecting different solvents into the metasurface‐embedded microfluidic channel, several distinct color images were obtained (Figure 5b,c). Nanofluidic hybrid metamaterials have been developed to achieve IR absorption and molecular sensing.^[^ ^242^ ^]^ Additionally, a mid‐infrared biosensor based on multi‐resonant metasurfaces placed in a polydimethylsiloxane (PDMS) microfluidic cell has been proposed to distinguish molecule‐specific information with high sensitivity.^[^ ^243^ ^]^ Although microfluidics have complex tuning and fabrication processes, they are potential candidates for realizing tunable metasurfaces in sensing applications and pixel‐size color‐transition displays.

#### 2.4.3 Stretchable Substrate‐Based Tunable Metasurfaces

Mechanically stretchable substrates can be employed to fabricate mechanically tunable metasurfaces in the visible frequencies. An ideal flexible substrate has a low refractive index and low Young's modulus to exhibit high elasticity and flexibility. Therefore, polymers, such as PDMS are suitable candidates for flexible substrates. A uniform array of TiO_2_ cylindrical dielectric resonators was embedded in a mechanically stretchable PDMS substrate to realize a mechanically tunable all‐dielectric metasurface at visible frequencies.^[^ ^248^ ^]^ Additionally, a mechanically tunable metasurface was developed to realize anomalous refraction by embedding Au nanorods on a PDMS substrate (Figure 5d).^[^ ^187^ ^]^ Hence, position‐dependent phase discontinuity can be tuned by stretching the substrate. In addition, a flexible dielectric metasurface was proposed to decouple optical properties and geometrical forms.^[^ ^249^ ^]^A structured fabric based on a chain mail architecture has been proposed to mechanically tune between soft and rigid states (Figure 5e).^[^ ^188^ ^]^

#### 2.4.4 Origami‐ and Kirigami‐Inspired Tunable Metasurfaces

Novel 3D fabrication techniques such as origami and kirigami have been researched recently based on the science of cutting and folding flat objects to realize adaptable 3D shapes. Mechanical tuning through origami‐ and kirigami‐based structures provides reconfigurable metasurfaces properties via structural configurations of constitutive unit cells. Accordingly, a kirigami‐based metasurface was proposed by varying the folding patterns of meta‐atoms. The proposed design was applied to realize reconfigurable metalenses and tunable anomalous refractors.^[^ ^246^ ^]^ Kirigami‐inspired metamaterials have been proposed for adaptable invisibility management.^[^ ^247^ ^]^ A reconfigurable origami metawall has been demonstrated for the tunable absorption and deflection of light, based on external mechanical stimuli (Figure 5f).^[^ ^34^ ^]^ Furthermore, a programmable mechanical metamaterial based on the 3D transformable kirigami strategy has been proposed with multiple degrees of freedom, which can evolve into over 0.3 million derived modules (Figure 5g).^[^ ^63^ ^]^ Further, a reconfigurable origami‐inspired acoustic waveguide that can dynamically manipulate acoustic waves based on externally applied deformation has been presented.^[^ ^250^ ^]^

**Table 1:** Summary of related works aimed to realize the tunable and reconfigurable metasurfaces by using different tuning mechanisms

Tuning mechanism	Materials	Operation regimes	External stimuli	Function	Ref.
Carrier doping	Semiconductors	THz to Visible	Optical	Modulator	[184]
			Optical	Absorber	[208]
			Electrical	Modulator	[244]
	Graphene	THz to NIR	Electrical	Phase modulator	[57]
			Electrical	OAM, beam steering	[21]
			Electrical	Modulator	[219]
			Optical	Absorber	[183]
	TCO	NIR to Visible	Optical	Modulator/polarizer	[206]
			Electrical	Absorber	[216]
Phase Transition	GST	THz to Visible	Electrical	Modulator/wavefront engineering	[199]
			Thermal	Beam switching	[245]
	VO_2_	THz to Visible	Electrical	Beam manipulation	[203]
			Optical	Absorber	[200]
			Thermal	Absorber	[202]
	Liquid Crystal	GHz to Visible	Electrical	SAR imaging	[212]
				Modulator	[213]
				Beam steering	[80]
Mechanical	MEMS	GHz to Visible	Electromechanical	Modulator	[238]
	Structural transformation	GHz to Visible	Mechanical	Anomalous reflection/lens	[246]
				Adaptive invisibility	[247]
	Microfluids	GHz to Visible	Microfluids	Polarization converter	[241]
Capacitance	PIN diodes	MHz to GHz	Electrical	Smart wireless power transfer	[220]
				Sum and difference beams	[221]
	Varactors	MHz to GHz	Electrical	3‐Bit RIS	[222]

### 2.5 Chemical Reactions to Realize Tunable Metasurfaces

The permittivity of a material can be tuned by modifying its optical properties via an electrochemical reaction. This is an effective and reversible fabrication method for tunable metasurfaces.

**Figure 6:**
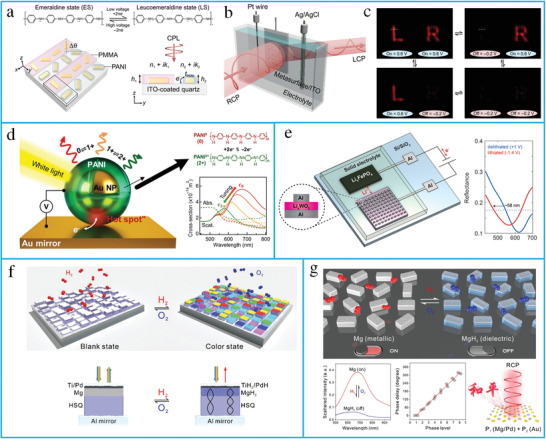
Chemically tunable metasurfaces. a) The conceptual illustration of electrochemically controlled metasurfaces working at visible frequencies. b) The experimental setup of in situ optimization of the metasurface performance. c) The holographic letters “L” and “R” are switched on and off. (a–c) reproduced with permission.^[^ ^251^ ^]^ Copyright 2021, the authors, published by American Association for the Advancement of Science. d) The schematic of scalable electrochromic nanopixels with optical scattering and absorption spectra. Reproduced with permission.^[^ ^252^ ^]^ Copyright 2019, American Association for the Advancement of Science. e) Structure of the solid‐state electrochromic device and measured reflection spectrum. Reproduced with permission.^[^ ^253^ ^]^ Copyright 2019, American Chemical Society. f) Dynamic color display using stepwise cavity resonators. By hydrogenation, the palette is changed from a blank state to a color state through the transition of Mg to MgH_2_. Reproduced with permission.^[^ ^189^ ^]^ Copyright 2017, American Chemical Society. g) Optical information encryption through dynamic metasurface holograms. Reproduced with permission.^[^ ^190^ ^]^ Copyright 2018, American Association for the Advancement of Science.

**Figure 7:**
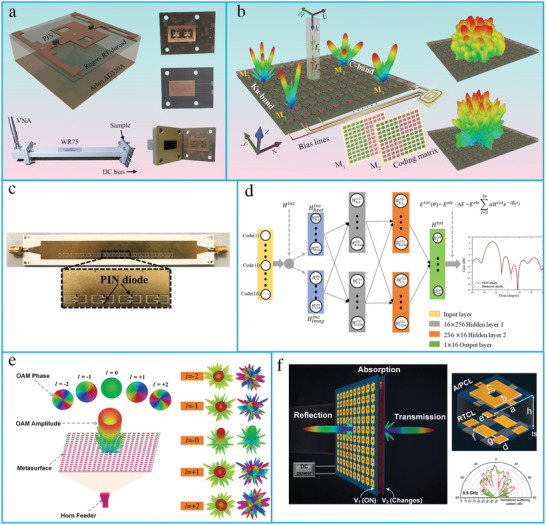
Space‐domain‐coding metasurfaces. a) The schematic of a 2‐bit dual‐band programmable metasurface unit cell. The fabricated samples and measurement setup in a waveguide environment. Reproduced with permission.^[^ ^46^ ^]^ Copyright 2021, The Optical Society. b) Programmable metasurface application for beam steering and diffusion‐like scattering. Reproduced with permission.^[^ ^260^ ^]^ Copyright 2021, IEEE. c) Structure of substrate‐integrated waveguide (SIW) based programmable metasurface. Reproduced with permission.^[^ ^261^ ^]^ Copyright 2020, IEEE. d) Physics‐inspired neural networks based on discrete dipole approximation. Reproduced with permission.^[^ ^265^ ^]^ Copyright 2022, IEEE. e) Transmissive programmable metasurface for high‐efficiency OAM generation. Simulated radiation patterns of different OAM beams. Reproduced with permission.^[^ ^19^ ^]^Copyright 2020, Wiley‐VCH. f) The conceptual illustration of multifunctional metasurface. The measured results of far‐field radiations. Reproduced with permission.^[^ ^266^ ^]^Copyright 2021, Wiley‐VCH.

A tunable metasurface based on lithium intercalation in vanadium pentoxide was developed. Owing to large variation in permittivity, several applications have been realized, such as handedness‐preserving reflectance, linear birefringence, and linear/circular dichroism.^[^ ^254^ ^]^ An electrochemically tunable metasurface based on polyaniline (PANI), a conducting polymer, has been proposed to realize high‐contrast switching in the visible region.^[^ ^251^ ^]^ When PANI was switched from its emeraldine state to a leucoemeraldine state by applying a voltage, a large variation in its refractive index was observed (**Figure** **6**a). Additionally, an electrochemically tunable metasurface was used to realize anomalous transmission and holography with a fast switching rate. An electrochemically driven color‐changing metasurface has also been proposed using a combination of conductive polymers and multilayered plasmonic architectures (Figure 6d).^[^ ^252^ ^]^ A solid‐state electrochromic device has been proposed using gap plasmon resonators filled with electrochromic WO_3_ (**Figure** **7**e). When lithium is inserted and removed from the solid‐state device, the optical properties of the Li_*X*_WO_3_ (0 <*X* < 0.2) dielectric are altered, thereby changing the resonant wavelength.^[^ ^253^ ^]^

Further, hydride‐loaded transition metals (e.g., Pd, Y, and Mg) possess better chemical reactivity than traditional plasmonic materials (e.g., Au, Ag, and Al). A metal‐to‐dielectric phase transition and large refractive index are achieved to realize tunable metasurfaces with diverse functionalities, including beam steering, active color display, holograms, and bifocal lenses. For example, magnesium‐based pixelated cavity resonators have been proposed for dynamic color printing (Figure 6f). By hydrogenation and dehydrogenation of magnesium, a metal‐to‐dielectric transition has been realized to enable black and colored states.^[^ ^189^ ^]^ Furthermore, a dynamic metasurface tuned by the hydrogenation and dehydrogenation of Mg nanorods has been proposed. The proposed metasurface was applied to tunable holograms and data encryption in the visible frequencies (Figure 6g).^[^ ^190^ ^]^ A dynamic metasurface also based on the hydrogenation and dehydrogenation of magnesium nanorods has been proposed to realize switchable singular beams at visible frequencies.^[^ ^255^ ^]^ Dynamic nanoantennas have been designed in the near‐infrared regime, tuned by modifying its permittivity via chemical redox reactions.^[^ ^256^ ^]^ The limitation of this tuning mechanism is the requirement of an environment with purified H_2_ and O_2_ flow, as well as a slow response time compared to other tuning techniques.

Additionally, since each tunable mechanism has its own advantages, such as (I) phase and amplitude modulation, (II) response time, (III) efficiency, (IV) power consumption, (V) operation regime, (VI) fabrication technology, and (VII) tunable functions, we have summarized related publications regarding tunable and reconfigurable metasurfaces using different tuning mechanisms (**Table** **1**).

## 3 Reconfigurable and Programmable Metasurfaces

Reconfigurable metasurfaces that can be programmed using an FPGA have received significant attention in recent years due to their ability to dynamically manipulate EM field and waves in real time. Digital metasurfaces characterize unit cells as digital bits with a value of “0” or “1.” In this section, we review the latest developments in space, time, and space–time‐modulated metasurfaces. Space‐gradient metasurfaces are constrained by the time‐reversal symmetry and Lorentz reciprocity, which can be resolved by introducing time‐modulated metasurfaces. These space–time‐modulated metasurfaces can realize novel physical phenomena and various applications, including overcoming the Lorentz reciprocity, harmonic manipulations, Doppler cloaks, and frequency conversion.

### 3.1 Space‐Gradient Metasurfaces

This section reviews space‐gradient metasurfaces designed to exhibit spatially varying responses, resulting in spatially varying phases, amplitudes, and polarizations of scattered fields. The generalized laws of refraction and reflection are used to describe anomalous reflection and refraction from a gradient metasurface.^[^ ^12^ ^]^ When light moves from one uniform medium to another, the reflection coefficients obey Snell's law: However, if a phase discontinuity is introduced at the interaction of the two mediums via a phase‐gradient metasurface, Snell's law needs to be revised by applying Fermat's principle. If a constant phase gradient is introduced along the interface, the refracted and reflected beams can move in arbitrary directions. Based on the response model, space‐gradient metasurfaces are classified as reflective, transmissive, waveguide‐type, and full‐space metasurfaces.

#### 3.1.1 Reflective Metasurfaces

Programmable reflective metasurfaces, also known as reconfigurable reflectarrays (RRAs), have received considerable attention due to their low profile, ease of fabrication, low complexity, and high‐gain beam scanning with reconfigurable characteristics. A dual‐frequency RRA has been designed for beam scanning applications with a good side‐loop level (SLL) and cross‐polarization performance at both frequencies.^[^ ^257^ ^]^ An electromagnetic reprogrammable coding metasurface has been developed to realize multiple holographic images in real time.^[^ ^258^ ^]^ Additionally, a programmable microwave imager has been presented by combining machine‐learning techniques with a 2‐bit coding metasurface. The proposed design can produce images and directly recognize objects without any the need for computational image reconstruction.^[^ ^74^ ^]^ A self‐adaptive smart metasurface has been developed to realize diverse functionalities without human interference.^[^ ^98^ ^]^ A dual‐frequency multibit programmable metasurface has also been proposed to control EM waves in real time.^[^ ^46^ ^]^ Further, an intelligent metasurface cloak driven by a deep learning algorithm that exhibits real‐time response to changing environments and incident waves without any human intervention has also been developed.^[^ ^97^ ^]^ An electronically reconfigurable metasurface has been proposed that operates in the microwave region to realize broadband Bessel‐like beams with a high efficiency.^[^ ^259^ ^]^ Saifullah et al. proposed a multibit programmable metasurface unit cell operating in the dual‐band.^[^ ^46^ ^]^ The proposed design was experimentally verified in a waveguide environment (Figure 7a). Furthermore, a 2‐bit programmable metasurface has been presented to realize beam steering and diffusion‐like scattering in the C and K bands (Figure 7b).^[^ ^260^ ^]^

#### 3.1.2 Radiative Metasurfaces

Radiative‐type metasurfaces are designed using arrays of complementary elements created on the conducting surface of a waveguide by coupling the energy from guided to free‐space waves. Each unit cell of the radiative metasurface is independently tunable and excites a guided wave that radiates part of the incident energy into free space. The resulting radiation pattern is an outcome of the contributions from all the unit cells; therefore, the field across the aperture can be controlled by tuning the response of each unit cell. Several mechanisms have been presented to excite metasurfaces, including waveguide‐fed cavity resonators and transmission lines, to realize reconfigurable radiating metasurfaces. A substrate‐integrated waveguide (SIW)‐based programmable metasurface has been presented to realize a dynamic pattern antenna.^[^ ^261^ ^]^ These dynamic metasurface antennas (MSAs) improve the resolution of SAR systems, while maintaining a large image size and higher signal‐to‐noise ratio (SNR).^[^ ^262^ ^]^ A low‐profile binary‐programmable metasurface has also been designed to realize the beamforming of leaky waves at a fixed frequency.^[^ ^263^ ^]^ Additionally, a dynamic metasurface has been incorporated in SAR imaging systems that can operate in spotlight and strip‐map modes at K‐band frequencies.^[^ ^264^ ^]^ Li et al. presented an SIW‐based programmable metasurface for a dynamic‐pattern antenna (Figure 7c).^[^ ^261^ ^]^ Such dynamic metasurfaces can achieve wide‐beam scanning and multi‐beam patterns in real time. Furthermore, an intelligent beamforming scheme based on a physics‐inspired neural network (PINN) and deep neural network (DNN) has been proposed, which can predict the code for desired patterns with more than 98.4% efficiency (Figure 7d).^[^ ^265^ ^]^

#### 3.1.3 Transmissive Metasurfaces

Transmitarrays, also known as flat lenses or lens arrays, have attracted substantial interest owing to their light weight, low profile, and low fabrication cost. Moreover, the lack of feed blockage compared to reflectarray antennas make them suitable candidates for modern wireless communication systems. Therefore, reconfigurable transmitarrays (RTAs) have attracted attention owing to their tunability and application prospects. An RTA unit cell can be realized as multilayer frequency‐selective surfaces (M‐FSSs),^[^ ^267^ ^]^ receiver‐transmitter structures,^[^ ^268^ ^]^ and coupled slots.^[^ ^269^ ^]^ A transmission‐type programmable metasurface for high‐efficiency OAM generation is presented (Figure 7e).^[^ ^19^ ^]^ Furthermore, a polarization‐rotation‐based 1‐bit RTA has been proposed, which has higher aperture efficiency and wider gain bandwidth.^[^ ^270^ ^]^

A 1‐bit wideband RTA was proposed based on the Vivaldi structure, phase shifter, and microstrip Yagi structure. A phase difference of 180° between the two states of the PIN diodes can be achieved using the current reversal mechanism.^[^ ^271^ ^]^An RTA has also been realized using two slot‐coupled patch antennas incorporated in a three‐layer structure operating at 5.5 GHz with 245° phase agility and 3 dB insertion loss.^[^ ^272^ ^]^ A Ka‐band electronically reconfigurable transmitarray, developed using PIN diodes, demonstrated a beam‐steering capability of ±60° with a radiation efficiency of 58%.^[^ ^273^ ^]^ An RTA antenna consisting of two orthogonal H‐shaped slots was also demonstrated in the Ku‐band. The proposed design achieved an aperture efficiency of 14%, beam scanning capability of ±50°, maximum gain of 17 dB, and 3 dB gain bandwidth of 9.6%.^[^ ^274^ ^]^

#### 3.1.4 Full‐Space

RTAs and RRAs have attracted significant attention for applications in radar and wireless communication due to their low profile, low cost, and high radiation gain. However, most of the presented designs could only control the transmitted or reflected wavefronts in half‐space. To overcome this limitation, a full‐space programmable metasurface that can achieve total transmission, total reflection, and total absorption in real time has been presented.^[^ ^275^ ^]^ A programmable metasurface has been proposed at microwave frequencies, which operates in the transmitted and reflected modes simultaneously for different polarizations incident waves.^[^ ^276^ ^]^ A reconfigurable multi‐functional metasurface has been proposed to realize transmission, reflection, and absorption simultaneously (Figure 7f).^[^ ^266^ ^]^ Dynamic control of transmission and reflection modulation for visible intensities has been demonstrated via an all‐dielectric phase‐change metasurface based on Fano resonances.^[^ ^277^ ^]^ A programmable metasurface has also been presented for polarization‐insensitive control of near/far‐field patterns in the reflection and transmission modes of EM waves.^[^ ^278^ ^]^ Additionally, a single‐layer circular ring slot element has been developed to realize a 1‐bit bidirectional reconfigurable transmit‐reflect array.^[^ ^279^ ^]^ Accordingly, a reflection–transmission (R–T) amplitude‐code‐based digital metasurface has been proposed for the full‐space control of EM waves.^[^ ^280^ ^]^

### 3.2 Time‐Modulated Metasurfaces

Reciprocity is a general concept that occurs in many areas of physics and engineering. The reciprocity theorem, as applied to circuits, states that any physical linear network, the positions of an ideal voltage source and an ideal ammeter can be interchanged without affecting their readings.^[^ ^281^ ^]^ Here, we present the Lorentz reciprocity theorem for electromagnetic fields which can be explained by using Maxwell's equations. The reciprocity theorem has many applications related to the transmitting and receiving properties of radiating systems.^[^ ^282^ ^]^ Let us assume two sets of sources $\left(\vec{J_{1}},\vec{M_{1}}\right),\left(\vec{J_{2}},\vec{M_{2}}\right)$) which generate the fields $\left(\vec{E_{1}},\vec{H_{1}}\right),\left(\vec{E_{2}},\vec{H_{2}}\right)$, respectively, in the volume *V* enclosed by the closed surface *S*.

When *S* encloses no sources, then $\vec{J_{1}}$ = $\vec{J_{2}}$ = $\vec{M_{1}}$ = $\vec{M_{2}}$ = 0, and the fields $\vec{E_{1}}$, $\vec{H_{1}}$ and $\vec{E_{2}}$, $\vec{H_{2}}$ are source‐free fields. In this case^[^ ^283^ ^]^

(1) $$\oint_{S}\vec{E_{1}}\times \vec{H_{2}}\cdot d\vec{S}=\oint_{S}\vec{E_{2}}\times \vec{H_{1}}\cdot d\vec{S}$$

When *S* is the inner surface of a perfectly conducting closed cavity, the result is

(2) $$\int_{V}\left(\vec{E_{1}}\cdot \vec{J_{2}}-\vec{H_{1}}\cdot \vec{M_{2}}\right)dv=\int_{V}\left(\vec{E_{2}}\cdot \vec{J_{1}}-\vec{H_{2}}\cdot \vec{M_{1}}\right)dv$$

This result is analogous to the reciprocity theorem of circuit theory. In other words, this result states that the system response $\vec{E_{1}}$ or $\vec{E_{2}}$ is not changed when the source and observation points are interchanged. That is, $\vec{E_{2}}$ (caused by $\vec{J_{2}}$) at $\vec{J_{1}}$ is the same as $\vec{E_{1}}$ (caused by $\vec{J_{1}}$) at $\vec{J_{2}}$.

**Figure 8:**
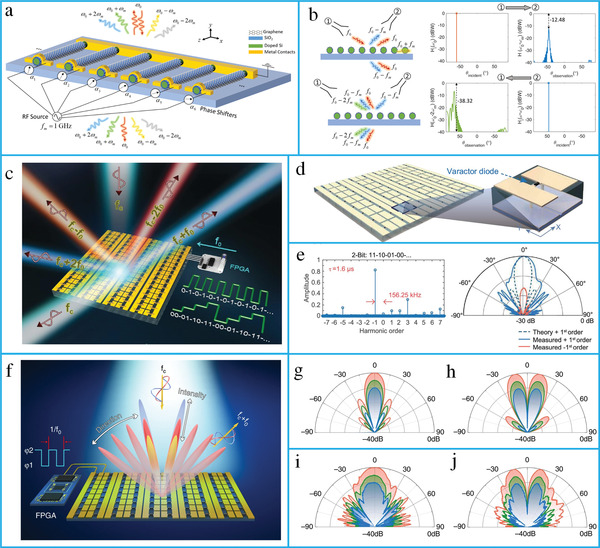
Time‐modulated metasurfaces. a) The conceptual illustration of a time modulated metasurface designed by graphene‐wrapped microwires. b) The nonreciprocal response of the time‐modulated metasurface. (a) and (b) reproduced under terms of the CC‐BY 3.0 license.^[^ ^118^ ^]^ Copyright 2018, the authors, published by IOP Publishing. c) The schematic of the time‐domain digital‐coding metasurface for new wireless communication systems. d) The schematic of the unit cell. e) The measured spectral intensities and H‐plane scattering patterns for the 2‐bit coding sequence. (c–e) reproduced under terms of the CC‐BY 4.0 license.^[^ ^89^ ^]^ Copyright 2018, the authors, published by Oxford University Press. f) The concept of the time‐domain digital coding metasurface that can control the harmonic amplitudes and phases independently. g,i) The simulated and measured results for the +1st order harmonic for the coding sequences of “00000000.” h,j) The simulated and measured results for the +1st order harmonic for the coding sequences of “00001111”. (f–j) reproduced under terms of the CC‐BY 4.0 license.^[^ ^197^ ^]^ Copyright 2018, the authors, published by Springer Nature.

The efficient and flexible control of harmonic conversion and breaking of reciprocity constraints are essential for many applications. Conventionally, phase shifters and other amplifiers were used to tailor harmonics, leading to high cost and difficulty in system integration. A time‐modulated metasurface based on modulation‐induced phase shift has been introduced to break reciprocity and generate higher‐order harmonics.^[^ ^118^ ^]^ The proposed metasurface has been designed using graphene‐wrapped microwires (**Figure** **8**a). Further, a time‐domain programmable metasurface has been demonstrated for the efficient manipulation of the spectral harmonic distribution (Figure 8c,d).^[^ ^89^ ^]^ Consequently, the measured spectral intensities and H‐plane scattering patterns for the 2‐bit coding sequence were analyzed (Figure 8e).

A limitation of the space–time metasurface is the coupling between the amplitudes and phases of various harmonics. Therefore, a time‐domain digital coding metasurface was proposed to independently control them. Beam scanning of multiple harmonics was achieved by tuning the phase of the metasurface via digital coding sequences instead of a phase‐shifting network (Figure 8f).^[^ ^197^ ^]^ When the metasurface is encoded with the sequence “00000000,” a directive beam along the normal direction is realized for the +1st order harmonic (Figure 8g,h). Decays of 0, 5, and 10 dB in magnitude were achieved by varying the biasing voltage. When the metasurface was encoded with the sequence “00001111,” two beams were realized in different angular directions (Figure 8i,j). Furthermore, a time‐domain coding metasurface‐aided wireless communication system has been presented for the direct modulation of baseband digital signals without conventional analog or digital circuits.^[^ ^284^ ^]^ A 2‐bit metasurface has been presented to realize a 4‐bit and higher phase by manipulating time‐coding sequences.^[^ ^285^ ^]^ Further, a discrete time‐invariant signal has been used in a time‐domain coding metasurface to demonstrate artificial Doppler shift for a Doppler cloak, useful for vehicle‐to‐vehicle communication.^[^ ^122^ ^]^

### 3.3 Space–Time Modulated Metasurfaces

**Figure 9:**
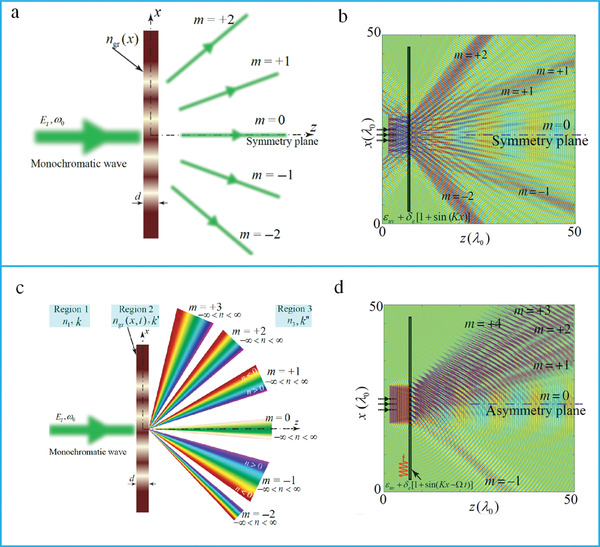
Diffraction from a transmissive grating. a) Conventional static diffraction grating with the same temporal frequency. b) FDTD simulation of a conventional time‐invariant grating. c) Transmissive space–time metasurface produces an asymmetric scattering and diffraction pattern where each spatial diffraction consists of the infinite number of temporal diffractions. d) FDTD simulation of a space–time periodic grating. (a–d) reproduced with permission.^[^ ^286^ ^]^ Copyright 2019, American Physical Society.

For linear and time‐independent systems, according to the electromagnetic Lorentz reciprocity theorem, the received and transmitted field ratios are equal in both the time‐forward and reversed propagation directions. Magneto‐optical media that can accomplish optical isolation can break this reciprocity.^[^ ^287^ ^]^ However, this approach is not suitable for system integration since bulky magnets must be used as an external bias. Nonlinear materials have been developed using the self‐biasing effect^[^ ^288^ ^]^ via an electric field, but nonreciprocity is power‐dependent. Thus, leaky‐wave antennas^[^ ^111^ ^]^ and spatiotemporally modulated waveguides^[^ ^109^ ^]^ have been employed to break reciprocity. Therefore, the space–time modulation mechanism has substantial use in metasurface platforms due to its compact size, improved integrability, and nonreciprocal behavior based on surface waves. By integrating the concept of time and space gradients in a metasurface, a nonreciprocal electromagnetically induced transparency (EIT) effect has been achieved.^[^ ^289^ ^]^ For conventional spatial diffraction gratings, symmetric diffraction patterns were observed with respect to the *x*‐axis (**Figure** **9**a). These gratings were restricted by the Lorentz reciprocity theorem. Finite‐difference time‐domain (FDTD) simulation of the conventional gratings shows that all the diffracted orders have the same wavelength (Figure 9b). For a space–time periodic (STP) diffraction grating, each spatial diffraction has an infinite temporal diffraction order (Figure 9c). The FDTD simulation results of the STP show that the diffraction orders are asymmetric with respect to the *x*‐axis and possess different wavelengths (Figure 9d).

**Figure 10:**
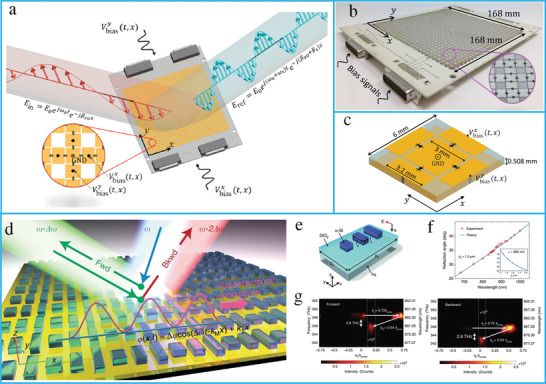
Space–time‐modulated metasurfaces. a) The conceptual illustration of spatially discrete, traveling‐wave modulation. b) The fabricated metasurface prototype. c) Schematic illustration of the metasurface unit cell. (a–c) reproduced with permission.^[^ ^290^ ^]^ Copyright 2020, American Physical Society. d) Conceptual illustration of nonreciprocal space–time metasurface. e) The schematic of unit cell. f) The calculated and measured anomalous reflection angles. g) Experimental illustration of nonreciprocal reflection. (d–g) reproduced under terms of the CC‐BY 4.0 license.^[^ ^291^ ^]^ Copyright 2019, the authors, published by Springer Nature.

**Figure 11:**
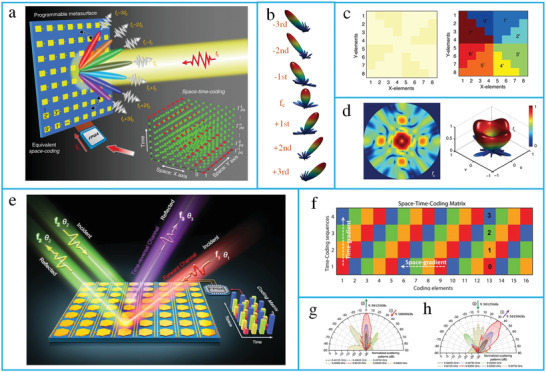
Space–time modulate metasurfaces. a) The concept of a space–time‐coding digital metasurface. b) 3D scattering patterns of harmonic beam steering. c) The amplitude and phase distributions. d) The 2D and 3D scattering patterns. (a–d) reproduced under terms of the CC‐BY 4.0 license.^[^ ^292^ ^]^ Copyright 2018, the authors, published by Springer Nature. e) The concept of space–time‐coding metasurface for nonreciprocal reflection. f) The space–time coding matrix. g) Measured scattering patterns for forward scenario. h) Measured scattering patterns for time‐reversal scenario. (e–h) reproduced with permission.^[^ ^293^ ^]^ Copyright 2019, Wiley‐VCH.

A spatiotemporally modulated metasurface has been demonstrated to realize nonreciprocity by radiating the incident beam in a far field and near‐field surface waves for forward and reverse scattering, respectively.^[^ ^101^ ^]^ Furthermore, a spatially discrete travelling‐wave modulated metasurface has been proposed (**Figure** **10**a).^[^ ^290^ ^]^ A space–time phase modulation metasurface based on resonating dielectric nanoantennas has been developed to achieve nonreciprocity (Figure 10d–g). To simultaneously control both the propagation direction and harmonic power distribution, space–time modulated metasurfaces have been proposed.^[^ ^114^ ^]^ Additionally, they can manipulate the spatial and spectral characteristics of EM waves (**Figure** **11**a).^[^ ^292^ ^]^ The proposed metasurface was applied to realize a vortex beam carrying an OAM (Figure 11c,d). Furthermore, a space–time coding digital metasurface has been presented to break time‐reversal symmetry and realize nonreciprocal reflections (Figure 11e).^[^ ^293^ ^]^ The metasurface consisted of 16 space‐coding elements and four time‐coding sequences (Figure 11f).

Additionally, a space–time coding digital metasurface has been presented to realize multifrequency beam steering and shaping.^[^ ^294^ ^]^ The proposed metasurfaces can be used in several applications, such as diffusion‐like scattering, multibeams, and OAM. Moreover, an amplifier‐based transmissive metasurface has been demonstrated for the nonlinear control of EM waves in the spatiotemporal domain. The proposed metasurface can break the Lorenz reciprocity owing to the nonreciprocity of unilateral amplification of the power amplifier.^[^ ^116^ ^]^ The most significant applications of space–time‐modulated metasurfaces are RISs. Recently, RISs have shown to be suitable candidates for 6G communication networks. In the next section, we describe the fundamental principles of RISs, framework for machine‐learning (ML)‐assisted RIS systems, and integration of RISs with emerging technologies.

## 4 RIS

With the recent advancement of metasurfaces design, RISs have been considered suitable techniques for smart and controllable radio environments. RISs are designed by integrating the reconfigurable elements as a 2D structure to realize passive beamforming with controllable intensity and direction. The propagation medium between transmitter and receiver has been considered a random entity as we cannot control the interaction of transmitted waves with the surrounding objects. RIS operates in a short range and can eliminate the transmitter RF chain; hence, it is more economical than the conventional multi‐antenna and relaying technologies. In the beginning, RISs was considered as hanging object on walls that could anomalously reflect incident signals in a different direction. However, RISs can be placed in the center of the communication environment to reflect and transmit the incident signals in desired directions.

### 4.1 RIS Principle and Hardware Design

**Figure 12:**
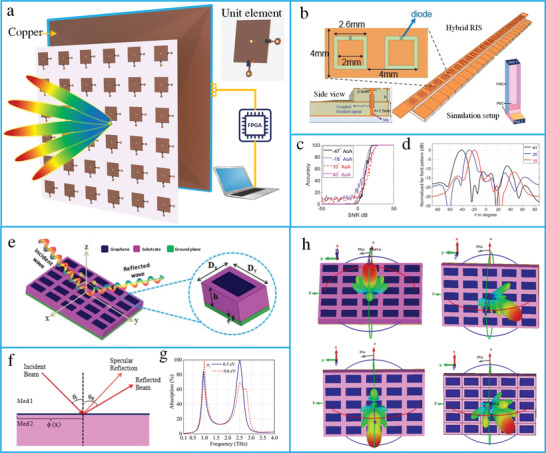
Design and principle of RIS. a) Architecture of RIS. b) The schematic of a hybrid RIS. c) Angle of arrival accuracy as a function of SNR. d) The far‐field pattern of three reflected beams. Reproduced under terms of the CC‐BY 4.0 license.^[^ ^294^ ^]^ Copyright 2021, the authors, published by Springer Nature. e) Graphene‐based RIS for active control of THz waves. f) The concept of anomalous reflection of RIS. g) Perfect absorption spectra of graphene‐based RIS. h) Beam steering using graphene RIS. Reproduced with permission.^[^ ^295^ ^]^ Copyright 2021, IEEE.

RISs are considered a promising technology for improving the spectrum and energy efficiency of wireless systems by the intelligent reconfiguration of the propagation environment of EM waves. RISs have been extensively researched due to their unique advantages in enhancing the wireless channel capacity. For wireless communication, RISs have been applied to realize anomalous reflection and beamforming. RISs are essentially planar surfaces designed with reconfigurable elements; each element contains top metallic patches, PIN diodes, and a dielectric substrate (**Figure** **12**a). The RISs are mounted with an FPGA to reconfigure the magnitude, phase, and polarization of each RIS element in real time. A hybrid metasurface with integrated reflecting and sensing characteristics has been proposed by coupling a portion of the incident wave to a waveguide (Figure 12b).^[^ ^294^ ^]^ The hybrid metasurface has been applied to realize anomalous reflections in the prescribed directions (Figure 12d). Furthermore, a graphene‐based RIS has been demonstrated to achieve dynamic control in the THz range in a wireless propagation environment (Figure 12e–h).^[^ ^295^ ^]^ The prototype was designed using rectangular graphene meta‐atoms placed on a silicon substrate, and copper was used as the ground layer. The proposed design was applied for tunable anomalous reflection, absorption, and THz communication.

### 4.2 RIS‐Based Wireless Communication

Future generations of wireless communications will include several novel applications, such as intelligent transportation systems, virtual reality, holographic projections, and brain–computer interfaces. Yuan et al. researched RIS‐empowered wireless communications and identified challenges and opportunities for future research.^[^ ^165^ ^]^ Pan et al. presented an RIS for 6G systems and demonstrated its principles, future directions, and applications.^[^ ^134^ ^]^ Alexandropoulos et al. presented an RIS for wireless communication for rich scattering and discussed the experimental results, opportunities, and challenges for next‐generation communication systems.^[^ ^296^ ^]^ Liu et al. demonstrated a smart radio environment scenario focused on the analysis of the challenges in commercializing and standardizing RISs from an industrial viewpoint.^[^ ^154^ ^]^ Lei et al. presented an RIS‐based symbiotic radio (SR) for ultra‐massive connectivity requirements in 6G and demonstrated its operating principles, application scenarios, implementation challenges, and opportunities for future research.^[^ ^129^ ^]^ Basharat et al. described an overview of RIS‐assisted 6G wireless networks and illustrated their practical implementation, deployment challenges, channel estimation, and future directions.^[^ ^128^ ^]^ Liu et al. presented a comprehensive survey of RISs with a focus on their operating principles, resource allocation, beamforming, and ML‐assisted wireless networks.^[^ ^133^ ^]^ Gong et al. demonstrated smart wireless communication based on IRSs with a focus on performance analysis, diverse applications, and future directions.^[^ ^181^ ^]^ An RIS‐assisted system has been proposed for robust beamforming design in the presence of a finite number of phase shifts at each element.^[^ ^297^ ^]^ To optimize the rate and energy efficiency of the RIS‐based communication system, the RIS phase shifts and number of reflecting elements have been jointly optimized.^[^ ^298^ ^]^ The ergodic capacity and outage probability performance of IRS‐based communication systems are enhanced by increasing the number of reflecting elements.^[^ ^299^ ^]^ The minimum number of reflecting elements required to maintain energy efficiency and spectral efficiency have been demonstrated.^[^ ^300^ ^]^ Numerical simulations of a large intelligent surface (LIS) demonstrated that inference suppression could be improved by increasing the number of terminals.^[^ ^146^ ^]^

**Figure 13:**
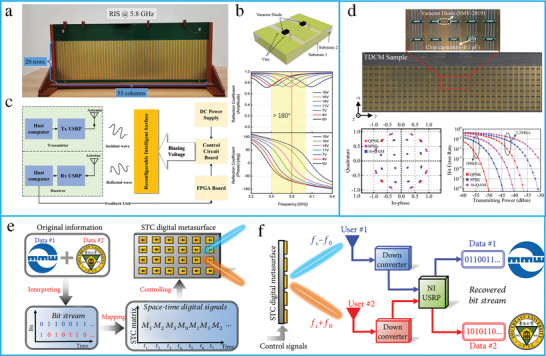
RIS aided wireless communication. a) The fabricated prototype of RIS. b) Schematic of RIS element, the phase and magnitude response of the RIS element. c) The system diagram of RIS aided communication system. (a–c) reproduced with permission.^[^ ^301^ ^]^ Copyright 2021, IEEE. d) The fabricated sample, bit error rates and measured constellation diagrams. Reproduced with permission.^[^ ^302^ ^]^ Copyright 2020, IEEE. Space–time coding metasurface for wireless communication. e) The transmitting process. f) The receiving process. (e) and (f) reproduced with permission.^[^ ^94^ ^]^ Copyright 2021, Springer Nature.

Since a majority of the recent research articles have been based on theoretical studies, experimental trials on RIS‐aided wireless communication are limited. Pei et al. proposed a prototype for RIS‐aided wireless communication (**Figure** **13**a–c).^[^ ^301^ ^]^ The proposed design was evaluated using indoor and outdoor field trials. Furthermore, a feedback‐based algorithm for adaptive beamforming has also been demonstrated, enabling smart reflections without modifying the communication standards. A multimodulation scheme based on a time‐domain digital coding metasurface (TDCM) has been presented for wireless communication (Figure 13d).^[^ ^302^ ^]^ Furthermore, a dual‐channel wireless communication system based on a 2‐bit space–time coding metasurface has been proposed (Figure 13e,f).^[^ ^94^ ^]^ The proposed system can simultaneously transmit two pictures to two designated users at different locations in real time.

### 4.3 Integrating RISs with Emerging Technologies for 6G Communication

The research discussed in the previous section demonstrates the usefulness of RIS‐aided wireless communication in improving the quality of service (QoS), channel capacity, and energy efficiency. In this section, we present the challenges and opportunities for the integration of RISs in 6G communication systems. The emerging technologies include RF localization, intelligent transportation, NOMA, cell‐free mMIMO, SWIPT, and UAV‐assisted wireless communication.

#### 4.3.1 RF Localization and Sensing

RISs have been proposed as potential candidates for smart radio environments and are consequently a promising solution for localization and sensing‐based services in future 6G networks.^[^ ^151^, ^152^, ^153^, ^154^ ^]^ Localization and sensing play important roles in the interaction of the digital and physical worlds, where intelligent devices are aware of their location as well as that of other devices. Notable applications include intelligent transportation systems, human‐to‐machine interfaces, and indoor and outdoor localization.^[^ ^303^ ^]^

##### Indoor Localization

Indoor localization has significant applications in airports, shopping malls, and smart factories, where accurate user positioning is important. Indoor localization accuracy is severely affected by the blockage of the line of sight (LoS). This limitation can be overcome by mounting the RIS on a wall and offering virtual LoS links. An RIS‐based multi‐user localization has been proposed in an indoor environment, and a localization protocol has been demonstrated for the coordination of AP, RIS, and users.^[^ ^304^ ^]^ Multiple RISs are deployed with single‐receive RF chains for user localization.^[^ ^305^ ^]^ The proposed design is based on the directional estimation of each RIS and maximum likelihood position estimation, which relies on the least‐squares line intersection. MetaRadar‐based multi‐user localization has been demonstrated via tunable metamaterial‐based reconfigurable radio reflection that can modify the radio environment.^[^ ^306^ ^]^

##### Intelligent Transportation Systems

RIS‐based vehicle‐to‐everything (V2X) communication^[^ ^156^ ^]^ and autonomous^[^ ^307^ ^]^ driving are envisioned as potential solutions for intelligent transportation systems. Autonomous vehicles must be capable of planning their movements in a dynamic environment shared with pedestrians and other vehicles.^[^ ^308^ ^]^ Wireless interconnected infrastructure, vehicles, and pedestrians can perceive environmental information and share it with nearby entities in real time.^[^ ^309^ ^]^ The V2X communication users can be considered to be distributing computing networks using computers and extended onboard sensors. Therefore, the potential dangers can be perceived in real time. With the implementation of RIS on billboards and buildings, the performance and accuracy of localization and sensing can be further improved.

#### 4.3.2 RIS‐Assisted Unmanned Aerial Vehicles

Unmanned aerial vehicles (UAVs) have attracted substantial attention for their applications in improving the performance of wireless communication due to their high maneuverability, low cost, and mobility.^[^ ^161^ ^]^ RIS‐enabled UAV communication systems are categorized as RIS‐assisted aerial base stations (BSs), RIS‐enabled UAV relays, and RIS‐assisted UAV UEs. In the first case, the RIS is mounted on buildings or billboards and UAVs act as aerial BSs. Therefore, the RIS provides a reconfigurable channel between the UAV and ground users. The secrecy rate of the RIS‐assisted UAV network was demonstrated by the joint optimization of the trajectory and power control of the UAV and phase distribution of the RIS.^[^ ^310^ ^]^ For RIS‐enabled UAV relay, the RIS is mounted on the UAV and acts as a passive relay. A UAV‐borne IRS has been demonstrated for wireless relay systems.^[^ ^311^ ^]^ To analyze the performance of the UAV‐IRS system, a mathematical framework for critical network parameters such as UAV altitude and number of IRS elements has been developed to maximize spectral and energy efficiency. UAV applications, such as UE, are assisted by ground BSs, whereas RISs can be used to improve the signal strength.

#### 4.3.3 RIS‐Aided Nonorthogonal Multiple Access

In non‐orthogonal multiple access (NOMA), the signal from multiple users is superimposed at different powers on the same frequency, and the desired information is decoded at the receiver via successive interference cancellation (SIC).^[^ ^329^ ^]^ Additionally, sum‐rate maximization has been proposed for a multicell IRS‐assisted NOMA network.^[^ ^330^ ^]^ An ML‐based approach has been proposed for phase shifter design and user partitioning in RIS‐enabled NOMA networks.^[^ ^331^ ^]^ The physical layer security (PLS) performance of the RIS‐aided NOMA network was also demonstrated in the presence of both internal and external eavesdropping.^[^ ^332^ ^]^ To improve the PLS of the network, a joint beamforming and power allocation scheme was proposed. The secrecy performance of the system can be further improved by increasing the number of reflecting elements in the RIS.

#### 4.3.4 RIS‐Assisted SWIPT

SWIPT is considered an effective technique for prolonging the energy demands of wireless communication networks, as it effectively carries energy and RF signal information simultaneously. Nevertheless, the low energy efficiency of the receiver is a critical issue in the practical implementation of SWIPT systems. To resolve it, RIS‐aided SWIPT is a promising solution.^[^ ^168^, ^169^, ^170^ ^]^ A RIS‐aided MIMO for SWIPT has been developed to improve the performance of information receivers (IRs) and energy receivers (ERs).^[^ ^323^ ^]^ IRS‐aided joint active and passive beamforming optimization has been demonstrated for SWIPT under QoS constraints.^[^ ^170^ ^]^ An IRS‐aided MISO system, which operates in the energy harvesting and signal‐reflecting phases, has been presented for throughput maximization.^[^ ^321^ ^]^ A self‐sustainable IRS‐aided wireless‐powered communication network for optimized energy and information relaying has also been developed.^[^ ^322^ ^]^

#### 4.3.5 Cell‐Free mMIMO

A cell‐free massive MIMO system consists of a large number of distributed access point antennas linked via a network controller, and each user can be served by all access points.^[^ ^333^, ^334^, ^335^ ^]^ An RIS‐based cell‐free MIMO communication system has been demonstrated, wherein multiple RISs and a single BS are deployed to serve multiple users.^[^ ^327^ ^]^ An energy‐efficient RIS‐based cell‐free MIMO communication system has been presented, wherein multiple BSs and RISs coordinate to serve numerous users.^[^ ^328^ ^]^ This method achieves a centralized hybrid beamforming design that increases the burden on the fronthaul network, as all APs need to share the users' channel with the CPU. Therefore, a decentralized beamforming scheme for IRS‐based cell‐free networks has been presented for locally updated beamformers.^[^ ^336^ ^]^ **Table** **2** summerizes the emerging technolgies that can be integrated with RIS to realize the future generation of wireless communications.

**Table 2:** Integrating RIS with emerging technologies

Description 1	Description 2	Description 3	Description 4	Ref.
RF localization and sensing	RSS based systems	MetaRadar localization system	Indoor localization of multi‐users	[306]
	Joint optimization of resources and RIS	V2X communications	Socially aware network with joint resource optimization	[156]
	Multi‐RIS‐assisted sensing system	Orthogonal matching pursuit based on ML approach	Single‐source positioning	[305]
	SISO	Cramer–Rao bounds	Outdoor localization	[312]
	MIMO‐OFDM system	Near‐ and far‐field propagation	3D localization in near‐field	[313]
	mmWave MIMO system	Channel modeling	Indoor and outdoor localization	[314]
RIS‐assisted UAV	UAV‐IRS mode	Outage probability and ergodic capacity	Maximize energy efficiency	[311]
	Aerial‐RIS to support UAV‐BSs	Jointly optimize RIS placement and phases of RIS elements	High energy efficiency	[315]
	RIS‐Assisted UAV	Bit error rate and outage probability	Coverage and performance	[316]
RIS‐aided NOMA	SISO‐RIS NOMA	Reflection coefficients of RIS and power allocation	Compare TDMA, FDMA, and NOMA for weighted sum rates	[317]
	MISO‐RIS‐NOMA	Active beamforming at BS and phase shifts of RISs	Joint optimization of phase shifts of RIS and power allocation	[318]
	MISO‐RIS‐NOMA	Active beamforming at BS and phase shifts of RISs	Beamforming optimization for the energy efficiency	[319]
	SISO‐RIS‐NOMA	Recourse allocation and reflection coefficients of RIS	Resource allocation and system throughput maximization	[320]
RIS‐assisted SWIPT	MISO	Multiple‐input single‐output	Throughput maximization	[321]
	Hybrid‐relaying	Hybrid‐relaying scheme	Sum rate maximization	[322]
	MIMO	Joint optimization at BS and phase shift of the IRS	Performance enhancement of information and energy receivers	[323]
RIS‐aided THz communication	Graphene‐based RIS	Reflection performance of graphene‐based RIS	Dynamic control of THz waves	[324]
	mMIMO	Channel estimation and compressive sensing	Holographic communication in the THz regime	[325]
RIS‐based cell‐free MIMO	mMIMO	Joint optimization of DL coefficients at AP and RIS phase shifts	Minimizing the information leakage to Eavesdropper	[326]
	MIMO	Hybrid beamforming	Sum rate maximization	[327]
	MIMO	Hybrid beamforming	Energy efficiency	[328]

### 4.4 RIS‐Aided Communication: An ML Perspective

ML techniques have received significant attention in wireless communications owing to their large search space and learning abilities. Deep learning (DL) and reinforcement learning (RL) have also been applied to RIS‐aided communication systems. RL and DL algorithms have been applied to diverse optimization scenarios, such as the secrecy rate of communication systems,^[^ ^337^ ^]^ aerial RIS,^[^ ^338^ ^]^ and user scheduling.^[^ ^339^ ^]^

#### 4.4.1 Deep Learning

Recently, deep learning (DL) has emerged as a powerful approach for solving communication system problems, including spectrum sensing^[^ ^340^ ^]^ and channel estimation.^[^ ^341^ ^]^ For RIS‐assisted communication systems, several approaches have been demonstrated, including ML, DL, RL,^[^ ^342^ ^]^ supervised learning,^[^ ^343^ ^]^ unsupervised learning,^[^ ^344^ ^]^ and federation learning. DL is an effective in RIS‐aided communication systems due to its powerful learning abilities.^[^ ^345^, ^346^, ^347^ ^]^ A deep learning‐based framework for channel estimation in LIS‐aided massive MIMO has been presented by designing a twin convolutional neural network (CNN).^[^ ^348^ ^]^

#### 4.4.2 Reinforcement Learning

Deep reinforcement learning (DRL) is an emerging solution for the optimization of RIS phase shifts without training labels and allows for online learning. A DRL‐based framework has been presented for phase‐shift modeling of a IRS‐aided MISO communication network.^[^ ^338^ ^]^ A DRL‐based RIS‐aided multiuser MISO system has also been demonstrated by the joint design of the PSM at the RIS to transmit the beamforming matrix to a base station.^[^ ^342^ ^]^ DRL‐based rate maximization using the RIS‐aided MISO wireless system has been presented, wherein half‐duplex and full‐duplex operating modes have been studied simultaneously.^[^ ^339^ ^]^ Additionally, a DRL‐based IRS has been demonstrated for secure wireless communication by the joint optimization of base‐station beamforming and IRS reflection beamforming.^[^ ^337^ ^]^

#### 4.4.3 Other ML Techniques

In addition to DL and DRL, several algorithms based on supervised learning, unsupervised learning, and federated learning techniques have been proposed to realize next‐generation communication systems. Therefore, these techniques are suitable for RIS‐aided wireless communication systems.

##### Supervised Learning

Owing to the fast convergence and low complexity of supervised learning algorithms, it can be applied to RIS‐aided communication systems with sufficient training data. A supervised learning algorithm has been demonstrated for RIS‐enhanced wireless communication systems, where a DNN was trained offline to devise a relation between the RIS phase configuration and the measured coordinate information.^[^ ^361^ ^]^

##### Unsupervised Learning

Unsupervised learning is not data‐hungry as it does not depend on prior knowledge, unlike supervised learning. Therefore, unsupervised learning algorithms^[^ ^362^ ^]^ can be applied in RIS‐aided wireless systems to overcome challenges such as channel state detection,^[^ ^363^ ^]^ beamforming,^[^ ^364^ ^]^ and transmission power control in device‐to‐device communications.^[^ ^365^ ^]^ An unsupervised learning algorithm has been proposed for passive beamforming in RIS‐aided wireless communication networks.^[^ ^344^ ^]^ Joint active and passive beamforming based on unsupervised learning have also been demonstrated for RIS‐based multi‐user MISO downlink systems.^[^ ^356^ ^]^ Unsupervised deep‐learning‐aided RIS has been demonstrated for broadcast communications in industrial IoT.^[^ ^160^ ^]^

##### Federated Learning

Federated learning (FL) has gained significant research interest in the area of distributed optimization and large‐scale ML because of its characteristics of statistical training models directly on remote devices or siloed data centers while keeping data localized. RIS‐assisted FL is a suitable candidate for integrating distributed learning and wireless communication systems. Over‐the‐air computation (AirComp)‐based FL has been presented for the fast model aggregation of IRS‐assisted communication systems.^[^ ^366^ ^]^ Over‐the‐air federated learning (AirFL) has been presented for multi‐RIS‐assisted communication systems to reduce aggregation errors and achieve faster convergence rates.^[^ ^359^ ^]^ Energy‐efficient communication has been proposed for IRS‐assisted communication systems, where FL has been applied to the energy‐consumption minimization problem.^[^ ^367^ ^]^ Therefore, FL algorithms can be applied in RIS‐assisted communication systems to overcome challenges such as privacy protection,^[^ ^368^ ^]^ channel estimation,^[^ ^358^ ^]^ and energy efficiency.^[^ ^360^ ^]^ **Table** **3** summerizes the related works aimed to realize the ML‐based RIS‐assisted communication.

**Table 3:** Summary of related works aimed to realize the ML‐based RIS‐assisted communication.

ML technique	Learning mechanism	Learning mechanism	Ref.
Deep learning	CDRN	Noise removal	[339]
	FNN	PHY key generation	[349]
Reinforcement learning	DDPG	Sum‐rate maximization	[331]
	PDS‐PER	Secrecy rate enhancement	[337]
	DDPG	Coverage rate maximization	[342]
	DDPG	Efficient resource allocation	[350]
	MDP	Sum‐rate maximization	[351]
Supervised learning	ODE‐based CNN	Performance maximization	[352]
	CNN	Sum‐rate maximization	[353]
	CV‐DnCNN	Performance maximization	[354]
	CNN	Achievable rate maximization	[355]
Unsupervised learning	RISBFNN	Gain enhancement	[344]
	CNN, FNN	Sum‐rate maximization	[356]
	DNN	Spectral efficiency	[160]
	NN	Throughput maximization	[357]
Federated learning	CNN	Channel estimation	[358]
	CNN	Propagation error reduction	[359]
	DNN	Energy‐efficient	[360]

## 5 STAR‐RIS

**Figure 14:**
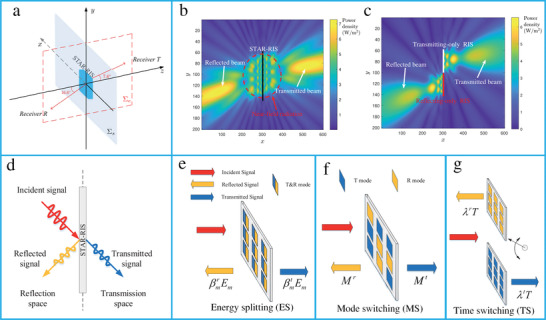
STAR‐RIS operating principle. a) The simulation setup of STAR‐RIS. b) The simulated radiation coverage of STAR‐RIS. c) The simulated radiation coverage of conventional RIS. (a–c) reproduced with permission.^[^ ^171^ ^]^ Copyright 2021, IEEE. d) The concept of STAR‐RISs. e–g) Three practical protocols for operating STAR‐RISs. (d–g) reproduced with permission.^[^ ^173^ ^]^ Copyright 2021, IEEE.

STAR‐RISs were introduced to overcome the half‐space operation of RISs. STAR‐RIS can manipulate both the reflected and transmitted signals, thereby achieving full‐space control of EM waves. STAR‐RISs are designed with several tunable elements that can simultaneously reconfigure the reflection and transmission coefficients. STAR‐RIS elements support both magnetic and electric currents to control the reflected and refracted signals. The differences between STAR‐RISs and conventional RISs are significant. For reflecting‐only RISs, the bottom of the element is covered with a metal plate, preventing the incident wave from penetrating the surface of the RIS to achieve better reflection. However, STAR‐RISs are designed without a metallic ground. Hence, the incident wave can penetrate the surface to achieve 360° coverage. Usually, STAR‐RISs have a more complex design, as the tunable elements need to control both the magnetic and electric currents. The reconfigurable characteristic is achieved via varactors or PIN diodes, and by varying the biasing voltage, the magnetic and electric reactance of each element can be adjusted. Several STAR‐RISs prototypes, such as transparent dynamic metasurfaces, have been implemented and presented to realize simultaneous reflection and transmission.^[^ ^369^ ^]^ Furthermore, a general STAR‐RIS hardware model has been proposed that can extend wireless coverage to 360° (**Figure** **14**a).^[^ ^171^ ^]^ The simulation results of the radiation patterns of the STAR‐RIS and conventional RIS were compared (Figure 14b,c). The desired angles of the reflected and transmitted signals were set as 16.6° and 7.6°, respectively. STAR‐RIS formed a beam‐like pattern, and the power densities were higher near the desired angles (Figure 14b).

### 5.1 Practical Operating Protocols

The incident signal on the surface of the STAR‐RIS is divided into reflected and transmitted signals (Figure 14d). To characterize the STAR features, a signal model with three protocols, namely energy splitting (ES), mode switching (MS), and time switching (TS), are used.^[^ ^173^ ^]^ For ES, the energy of the incident signal is divided as the entire surface operates in the transmission and reflection modes simultaneously. A higher degree of freedom is achieved via ES as the transmission and reflection coefficients of each unit element can be optimized (Figure 14e).

For MS, the STAR‐RIS elements are divided into R‐ and T‐mode operating groups (Figure 14f). This STAR‐RIS can be considered a transmit‐only RIS and reflect‐only RIS of reduced size. The drawback of MS is that it cannot match the reflection and transmission beamforming gain of ES because all the elements are not working in both the reflection and transmission modes simultaneously. However, the “on–off” switching operating protocols of MS are less complex and can be easily implemented. STAR‐RIS based on TS can switch the elements between the R and T modes at different times (Figure 14g). A balance between front‐ and back‐side communication can be realized by optimizing the time slots for transmitting and reflecting signals. TS is advantageous compared with ES and MS because the transmission and reflection coefficients are not coupled for a given time slot, and can hence be optimized independently. For TS, periodic time switching requires strict time synchronization, thereby increasing the implementation complexity compared to ES and MS.

### 5.2 STAR‐RIS Hardware Prototype and Experimental Evaluation

**Figure 15:**
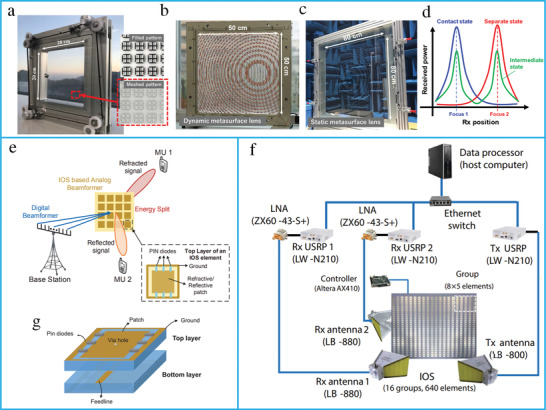
STAR‐RIS hardware prototype and experimental evaluation. a) Fabricated transparent dynamic metasurface. b) The prototype of dynamic metasurface lens. c) Fabricated sample of static metasurface lens. d) Design concept for FZP theory‐based dynamic metasurface lens. (a–d) reproduced with permission.^[^ ^369^ ^]^ Copyright 2021, The Optical Society. e) The concept of intelligent omni surface (IOS) assisted hybrid beamforming. f) The concept of an IOS‐aided wireless network. g) Schematic of an IOS element. (e–g) reproduced with permission.^[^ ^370^ ^]^ Copyright 2022, IEEE.

Published research on hardware prototypes and experiments on STAR‐RISs is limited. In this section, the hardware implementation of STAR‐RISs is presented. A transparent dynamic metasurface has been demonstrated for the simultaneous control of transmission and reflection, designed using transparent glass as a substrate to realize optical transparency (**Figure** **15**a).^[^ ^369^ ^]^

Dynamic switching between reflection and transmission is realized by stacking the substrate onto another substrate and controlling the gap between them (Figure 15b,c). Furthermore, IOS‐assisted hybrid beamforming of two users has been demonstrated (Figure 15e).^[^ ^370^ ^]^ When the signal is incident on the IOS, a fraction of the signal is refracted to the other side of the surface, while another fraction is reflected toward the same side. The IOS prototype consists of 640 elements (Figure 15f), and the state of the IOS element can be varied via preloading in the FPGA. The prototype IOS unit element consists of two PIN diodes on the top metallic layer, substrate, and ground (Figure 15g). The IOS element can realize four states by switching the two PIN diodes ON and OFF.

### 5.3 STAR‐RIS Applications

STAR‐RISs use passive reflecting and transmitting elements integrated on a planar surface to control EM wave propagation in full‐space. STAR‐RIS has reconfigurable characteristics both in reflection and refraction; therefore, it offers more degrees of freedom (DoFs) for the flexible manipulation of signal propagation. Optically transparent STAR‐RISs are well‐suited for existing building structures; therefore, they do not have any undesired aesthetic effects.^[^ ^369^ ^]^ Hence, they can be easily deployed to replace windows in the existing structure. A STAR‐RIS is more environment‐friendly and energy‐efficient than conventional relaying systems since it manipulates signal propagation by reconfiguring the phase modulation of each unit cell.

#### 5.3.1 Full‐Space Coverage

**Figure 16:**
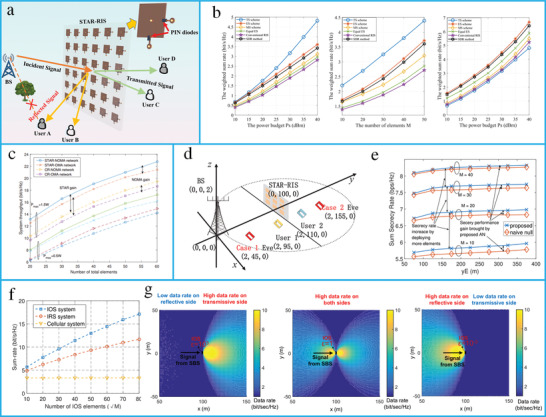
STAR‐RIS applications. a) Concept of STAR‐RIS aided communication. b) The optimized sum‐rate of STAR‐RIS‐aided MIMO system. Reproduced with permission.^[^ ^374^ ^]^ Copyright 2022, IEEE. c) The relation of sum rate with the surface size. Reproduced with permission.^[^ ^379^ ^]^ Copyright 2021, IEEE. d) Simulation setup of secrecy performance of STAR‐RIS‐assisted NOMA. e) The comparison of secrecy performance. (d) and (e) reproduced with permission.^[^ ^380^ ^]^ Copyright 2022, IEEE. f) The relation of sum rate with the surface size. g) Simulation of the maximum data rate with different ϵ. Reproduced with permission.^[^ ^381^ ^]^ Copyright 2022, IEEE.

STAR‐RISs can achieve full‐space control of EM waves; therefore, they can serve users on both sides of the surface. If deployed at the end of a cell, the user within the cell coverage and beyond can be served due to the simultaneous reflection and refraction. Compared to IRS, where the mobile unit can receive only the reflective signal, STAR‐RIS can receive both reflected and transmitted signals (**Figure** **16**a). A finite phase shift in the IOS‐assisted system has been proposed based on a branch‐and‐bound‐based algorithm. The downlink spectral efficiency of the MU can be maximized based on IOS phase‐shift optimization. The simulation results showed that the IOS‐assisted communication system had significantly enhanced coverage efficiency compared to reflect‐only RIS‐assisted systems.^[^ ^371^ ^]^ Furthermore, a STAR‐RIS‐aided two‐user communication network has been demonstrated, and the fundamental coverage range of the system has been characterized.^[^ ^372^ ^]^ For both OMA and NOMA, sum coverage range maximization has been demonstrated for the STAR‐RIS. Moreover, the reflection and transmission coefficients and resource allocation at the access point are jointly optimized.

#### 5.3.2 Interference Cancellation

Owing to the dense deployment of cells in future mobile communication systems, several users can operate within the coverage range of several cells. Such a scenario can increase multi‐cell interference and degrade the performance of the network. STAR‐RISs can direct the signal to a definite user on one side of the surface, thereby eliminating interference toward the user on the other side of the surface and alleviating multi‐cell interference.

#### 5.3.3 Physical Layer Security

In the presence of unauthorized eavesdroppers in a wireless medium, confidential messages can be sent through a PLS without higher‐layer encryption. The implementation of PLS for secure communication can be improved using a non‐reciprocal channel created by the STAR‐RIS. Accordingly, users on one side of the STAR‐RIS can receive the reflected signals, and eavesdroppers on the opposite side receive only the noise generated by the refracted signal.

#### 5.3.4 Sensing and Localization

Sensing and localization have several applications, including drone tracking, personal radar, and emergency‐call localization.^[^ ^373^ ^]^ However, the received signals at different locations must be distinguishable for better localization accuracy. The STAR‐RIS can tailor the propagation channels and enhance these differences; therefore, it is a promising solution for RF sensing and localization.

### 5.4 STAR‐RIS Aided Wireless Communications

#### 5.4.1 Sum Rate Optimization

Weighted sum rate (WSR) optimization in a STAR‐RIS‐aided MIMO system has been proposed based on the ES scheme.^[^ ^374^ ^]^ For unicast communication, the WSR increases with Ps for all schemes (Figure 16b). However, TS performs better than ES and MS as it can eliminate interuser interference. By increasing the surface size of the STAR‐RIS, better signal strength can be achieved, and hence, the WSR has a larger surface area. For broadcast communication, ES and MS perform better than TS. Furthermore, joint beamforming and TRCs are optimized for a STAR‐RIS‐aided MISO network considering the discrete coefficient.^[^ ^375^ ^]^

#### 5.4.2 Channel Estimation

Uplink channel estimation for the STAR‐RIS‐based two‐user wireless communication system has been presented.^[^ ^376^ ^]^ A practical coupled phase‐shift model based on the ES protocol was devised for joint channel estimation of both users. Furthermore, channel model approximation and performance analysis of the STAR‐IOS‐assisted NOMA network have been presented.^[^ ^377^ ^]^

#### 5.4.3 Coverage Characterization

In contrast to RISs, which can reflect the incident signal, STAR‐RISs can send signals to the MU in both a transmissive and reflective format and enhance the wireless coverage. A coupled phased shift model for two user scenarios is considered, where the AP transmits the information to both users that are present on both sides of the STAR‐RIS.^[^ ^371^ ^]^ The diversity gains of conventional RIS and STAR‐RIS are compared. Further, the coverage characterization of STAR‐RIS‐assisted communication for OMA and NOMA has been presented.^[^ ^378^ ^]^ Resource allocation in multi‐career communication based on OMA and NOMA has also been presented. It was concluded that same‐side user pairing is preferable in OMA; however, the transmission‐and‐reflection scheme provided better results with NOMA. The STAR‐RIS‐aided NOMA performed better than the OMA and conventional RIS (Figure 16c).^[^ ^379^ ^]^

#### 5.4.4 STAR‐RIS Enabled NOMA Communication

NOMA has gained considerable attention as a potential candidate for next‐generation multiple access (NGMA) since it can support massive connectivity and enhance spectrum efficiency. The achievable sum rate of the STAR‐RIS‐aided NOMA network is maximized by the joint optimization of the decoding order, active beamforming, power allocation coefficients, transmission, and reflection beamforming.^[^ ^382^ ^]^ The outage performance of the STAR‐RIS‐assisted NOMA network can be analyzed by randomly deployed users, and the simulation results showed that the STAR‐RISs could improve the channel quality for its users.^[^ ^383^ ^]^ For simultaneous intercell interference cancellation and signal enhancement, a STAR‐RIS‐aided NOMA network has been demonstrated.^[^ ^384^ ^]^ The proposed design can outperform conventional signal‐enhancement‐based (SEB) and signal‐cancellation‐based (SCB) designs.

#### 5.4.5 Secrecy Performance

A combination of STAR‐RISs and NOMA can significantly enhance coverage; however, eavesdroppers may have the same advantages. To overcome this and maximize secrecy, an artificial noise (AN) secure communication approach has been proposed (Figure 16d).^[^ ^380^ ^]^ The secrecy performance of the proposed method was compared with that of the baseline naive null scheme (Figure 16e). Further, the secrecy performance of a MISO network based on STAR‐RIS was presented by the joint optimization of beamforming, and transmission and reflection coefficients that can maximize the weighted sum secrecy rate (WSSR).^[^ ^172^ ^]^ Additionally, an IOS‐aided secure MIMO communication network has been demonstrated for a multiantenna eavesdropper scenario.^[^ ^385^ ^]^

#### 5.4.6 STAR‐RISs for Cellular Networks

STAR‐RISs play a significant role in the coverage extension of a communication system in the presence of obstacles between users and BSs or APs. For outdoor communication scenarios, STAR‐RIS can offer a substitute link between the BS and user by mounting STAR‐RISs on billboards and building facades. Transparent STAR‐RISs can be mounted on the body of vehicles, airplanes, and cruise ships to increase signal strength by using the refractive characteristics. Additionally, an IOS‐based multi‐user communication system has been proposed to improve the QoS of many MUs presented on both sides of the surface.^[^ ^381^ ^]^ By increasing the number of IOS elements, the average sum rate increases (Figure 16f). When the ISO operates in both reflective and refractive modes, the sum rates on both sides of the surface are improved. In reflective‐only mode, most of the power is reflected on the left side. In the refractive‐only mode, power is transmitted on the right side of the surface (Figure 16g).

#### 5.4.7 STAR‐RISs Assisted Indoor Communication

STAR‐RISs can act as a bridge for outdoor‐to‐indoor communication due to simultaneous transmission and reflection characteristics. As STAR‐RISs provide full‐space coverage, it can decrease the distance by avoiding the multihop bounces of the signal to reach the user, therefore, improving the received signal power. STAR‐RIS‐based indoor communications are presented, where STAR‐RIS can be mounted on the walls to improve the QoS of indoor communication systems. Furthermore, in order to eliminate the blind spots of wireless fidelity (WiFi) networks and visible light communications, a link between the users and APs is formed through STAR‐RIS to enhance the coverage.

#### 5.4.8 STAR‐RISs Aided Communication: An ML Perspective

Federated learning (FL), a distributed machine‐learning framework, is a promising technique that relies on decentralized data and protects user privacy.^[^ ^386^ ^]^ A unified framework of NOMA and federated learning based on STAR‐RIS has been presented to attain improved performance compared to benchmarks.^[^ ^387^ ^]^

## 6 Conclusions and Perspectives

We have presented a comprehensive review of the recent advances in intelligent metasurfaces, focusing particularly on tuning mechanisms, hardware design, and their diverse applications. We have discussed reconfigurable and programmable metasurfaces, classified into space‐gradient, time‐modulated, and space–time‐modulated metasurfaces. Moreover, we have described the fundamental principles of RISs, ML‐powered RIS systems, and integration of RIS with developing technologies for 6G wireless networks. Finally, we have presented a comparison between conventional reflect‐only RISs and STAR‐RISs with respect to their physical mechanism, hardware design, and promising applications.

Reconfigurable metasurfaces based on PCMs offer excellent phase and amplitude modulation. Nevertheless, their switching speed was comparatively slow (<1 kHz) and device efficiency was moderate. The most commonly used PCMs are GST and VO_2_ that exhibit non‐volatile and volatile switching, respectively. Notable applications include beam steering, optical switching, thermal modulation, bifocal lenses, and holographic imaging. Graphene‐based tunable metasurfaces have also been used to realize ultrathin devices with high‐speed operation, large phase modulation, and low power consumption. Such devices can operate in the THz and MIR regimes. Prominent applications include beam steering, phase modulators, and tunable absorbers. Electrically tunable transparent conducting oxide metasurfaces have received significant interest for their operation in the NIR and MIR regimes. Such a metasurface design offers large refractive index modulation, excellent phase modulation, low power consumption, and high operation speed. Prominent applications include tunable absorbers, metalenses, and beam steering. LCs are a promising solution for realizing tunable metasurfaces as they exhibit complete 2π phase modulation and outstanding amplitude modulation. Such tunable metasurfaces have low power consumption and high efficiency and can operate in the microwave, visible, and NIR regimes. Notable applications include light modulation, color filtering, and beam steering. The most popular tuning mechanism for realizing reconfigurable intelligent surfaces at microwave frequencies is lumped elements. PIN diodes and varactors are also commonly used, and a DC bias is applied to tune their impedance and realize reconfigurable functions.

Mechanical tuning through origami‐ and kirigami‐based structures provides reconfigurable properties to metasurfaces by the structural configuration of constitutive unit cells. Additionally, MEMS are suitable candidates for realizing active metamaterials owing to their reconfigurable mechanical structures, low power consumption, and compatibility with CMOS technology. Microfluidics has also been applied to active mechanical metamaterials, specifically for biosensing applications. Microfluid‐based tunable metasurfaces exhibit substantial amplitude modulation and a device efficiency of 55–95%. However, they have a slow response time, usually on the millisecond scale. Mechanically‐stretchable substrate‐based tunable metasurfaces exhibit large amplitude modulation, large phase modulation, and high device efficiency. However, they have slow response times, are environment‐sensitive, and cannot control each unit element individually. Mechanically stretchable substrates have been employed to realize tunable metasurfaces in the visible to NIR regime, and their notable applications include color filters and metalenses. The permittivity of a material can be tuned by modifying its optical properties via an electrochemical reaction. Chemically tunable metasurfaces have a phase modulation of 180°, low modulation speed (<0.1 Hz), and device efficiency of up to 75%. Prominent applications include holograms, beam steering, and dynamic color filters. Each tuning mechanism has unique characteristics; therefore, the selection of the tuning mechanism depends on the desired functionality of the device. For example, if applications require a high modulation speed (>kHz), electrical tunability is ideal. However, they have low efficiency and are limited to a certain operational wavelength range. Conversely, LC‐based active metasurfaces have high efficiency and a large operational spectral range; however, they have slow modulation speeds (<1 kHz).

Programmable metasurfaces^[^ ^69^, ^70^, ^71^, ^72^, ^73^ ^]^ have been developed by embedding an FPGA to dynamically manipulate the EM wave and enable switching between diverse functions in real time by varying the input coding sequences. Accordingly, space‐gradient metasurfaces are constrained by time‐reversal symmetry and Lorentz reciprocity, which are overcome by introducing a temporal gradient metasurface. Moreover, spatiotemporal metasurfaces have been that can realize novel physical phenomena and applications have been developed, including breaking the Lorentz reciprocity, harmonic manipulations, Doppler cloaks, and frequency conversion. The functionalities of tunable and reconfigurable metasurfaces can be enhanced by adding sensors to detect temperature, humidity, illuminating light, etc., thus paving the way for intelligent metasurfaces. These intelligent metasurfaces offer intelligent control of EM waves, leading to the development of numerous novel devices and applications in various frequency regimes. Additionally, they are equipped with sensing and feedback components to control the reprogrammable functions without human intervention.

While we have already witnessed substantial progress in the field of tunable and intelligent metasurfaces, several challenges and applications require further exploration. For example, the independent control of amplitude and phase is required for the dynamic control of EM waves. For the optical frequency band, local tuning mechanisms should be further explored as the GHz/THz regime‐tuning methods are not compatible with optical frequencies. From a material perspective, novel materials with tunable characteristics that are compatible with CMOS technology must be explored. At present, the space–time coding metasurface is phase‐modulated only. Hence, the development of amplitude modulation and amplitude‐phase modulation can offer more practical and flexible control of EM waves. Furthermore, most STC digital metasurfaces are reflect‐only, limiting feed blockage; therefore, transmission‐type and waveguide‐fed metasurfaces must be researched in the future. STC digital metasurfaces based on PIN diodes or varactors have limitations in terms of switching speed and are only applicable in the microwave band. The use of different materials such as indium tin oxide (ITO), graphene, and phase‐changing materials to realize STCs in the THz and optical frequency regimes is needed.

### 6.1 Novel Materials with Tunable Characteristics

To further improve the performance and sustainability of tunable and intelligent metasurfaces, novel active materials are required. These materials include novel conducting oxides, and phase‐change and 2D materials. Therefore, extensive material research is required to explore materials providing large refractive index variations upon external stimuli, lower response times, and compatibility with existing nanofabrication techniques. Another challenge, specifically for optical metasurfaces, is the fabrication of a large‐scale metasurface. Since metasurfaces are fabricated via expensive techniques such as electron beam lithography, sustainable mass production of large‐area metasurfaces is difficult. A separately controllable unit cell with a distinct optical response is also necessary for many applications. Therefore, their development is important for realizing more practical and sustainable devices.

### 6.2 Sustainable Development

Although electronic system design and fabrication have developed rapidly in recent years, several significant problems are prevalent when designing sustainable electronics, including the reduction of the environmental impact of e‐waste and environmentally friendly materials without compromising the performance. Therefore, significant attention should be diverted toward reducing the environmental impact of production processes, materials, and consumption. The material fabrication process consumes a large amount of energy and is a substantial source of greenhouse gas (GHG) emissions, producing ≈25% of all anthropogenic CO_2_ emissions. This waste is generated during both production and disposal. Therefore, improving the fabrication process and efficient use of materials can contribute toward sustainability and environmental benefits.^[^ ^388^ ^]^ Tunable and reconfigurable metasurfaces also play an important role in sustainable development, as a device, once designed and fabricated, can be reconfigured and reprogrammed for various applications. Since refabrication increases energy consumption and produces more CO_2_, reusing the reconfigurable metasurface for new applications is environmentally friendly.

### 6.3 Green Communications Toward 6G

In the current 5G and future 6G eras, as the network infrastructure grows rapidly, the number of network nodes will increase exponentially, leading to rising energy costs and increasing carbon footprint. Researchers have proposed two solutions to reduce the energy consumption in 6G: energy harvesting^[^ ^389^ ^]^ and energy‐efficient network‐management algorithms.^[^ ^390^ ^]^ RF harvesting not only realizes SWIPT but also employs an interference signal. For energy‐efficient network management, AI can be adopted to simplify the traditional mathematical iteration process. Further, RISs and STAR‐RISs can be employed to realize SWIPT and improve the energy efficiency of traditional communication systems. In addition, to realize AI‐based green communication, the energy consumption of AI algorithms should be investigated.^[^ ^391^ ^]^ Although recent research has focused on performance improvement compared to conventional algorithms, the energy requirement for training and running the AI models has been typically neglected. Therefore, the minimization of energy requirement is key to developing AI‐based green communication in future research, urging the design and execution of AI algorithms in an energy‐efficient manner.

## Conflict of Interest

The authors declare no conflict of interest.

## References

[1] V. G. Veselago , Usp. Fiz. Nauk 1967, 92, 517.

[2] J. B. Pendry , A. J. Holden , D. J. Robbins , W. Stewart , IEEE Trans. Microwave Theory Tech. 1999, 47, 2075.

[3] R. A. Shelby , D. R. Smith , S. Schultz , Science 2001, 292, 77. 11292865 10.1126/science.1058847

[4] R. M. Walser , in Complex Mediums II: Beyond Linear Isotropic Dielectrics, Vol. 4467, SPIE, Bellingham, WA 2001, pp. 1–15.

[5] S. B. Glybovski , S. A. Tretyakov , P. A. Belov , Y. S. Kivshar , C. R. Simovski , Phys. Rep. 2016, 634, 1.

[6] C. L. Holloway , E. F. Kuester , J. A. Gordon , J. O'Hara , J. Booth , D. R. Smith , IEEE Antennas Propag. Mag. 2012, 54, 10.

[7] D. R. Smith , J. B. Pendry , M. C. Wiltshire , Science 2004, 305, 788. 15297655 10.1126/science.1096796

[8] X. Bai , F. Zhang , L. Sun , A. Cao , C. He , J. Zhang , W. Zhu , Nanophotonics 2022, 11, 1389. 10.1515/nanoph-2021-0790 PMC11502101 39634612

[9] S. Liu , T. J. Cui , L. Zhang , Q. Xu , Q. Wang , X. Wan , J. Q. Gu , W. X. Tang , M. Qing Qi , J. G. Han , Adv. Sci. 2016, 3, 1600156. 10.1002/advs.201600156 PMC5096125 27840801

[10] Z. Wang , S. Li , X. Zhang , X. Feng , Q. Wang , J. Han , Q. He , W. Zhang , S. Sun , L. Zhou , Adv. Sci. 2020, 7, 2000982. 10.1002/advs.202000982 PMC7539192 33042739

[11] Y. Hu , X. Ou , T. Zeng , J. Lai , J. Zhang , X. Li , X. Luo , L. Li , F. Fan , H. Duan , Nano Lett. 2021, 21, 4554. 34047184 10.1021/acs.nanolett.1c00104

[12] N. Yu , P. Genevet , M. A. Kats , F. Aieta , J.‐P. Tetienne , F. Capasso , Z. Gaburro , Science 2011, 334, 333. 21885733 10.1126/science.1210713

[13] I. Kim , W.‐S. Kim , K. Kim , M. A. Ansari , M. Q. Mehmood , T. Badloe , Y. Kim , J. Gwak , H. Lee , Y.‐K. Kim , Sci. Adv. 2021, 7, eabe9943. 33827821 10.1126/sciadv.abe9943 PMC8026120

[14] F. Zhang , M. Pu , P. Gao , J. Jin , X. Li , Y. Guo , X. Ma , J. Luo , H. Yu , X. Luo , Adv. Sci. 2020, 7, 1903156. 10.1002/advs.201903156 PMC7237853 32440472

[15] Y. Zhai , H.‐S. Kwon , B.‐I. Popa , Phys. Rev. Appl. 2021, 16, 034023.

[16] A. M. Wong , G. V. Eleftheriades , Phys. Rev. X. 2018, 8, 011036.

[17] Z. Li , E. Palacios , S. Butun , K. Aydin , Nano Lett. 2015, 15, 1615. 25664815 10.1021/nl5041572

[18] S. Sun , K.‐Y. Yang , C.‐M. Wang , T.‐K. Juan , W. T. Chen , C. Y. Liao , Q. He , S. Xiao , W.‐T. Kung , G.‐Y. Guo , Nano Lett. 2012, 12, 6223. 23189928 10.1021/nl3032668

[19] X. Bai , F. Kong , Y. Sun , G. Wang , J. Qian , X. Li , A. Cao , C. He , X. Liang , R. Jin , Adv. Opt. Mater. 2020, 8, 2000570.

[20] Y. Shuang , H. Zhao , W. Ji , T. J. Cui , L. Li , IEEE J. Emerg. Sel. Top. Circuits Syst. 2020, 10, 29.

[21] K. Rouhi , H. Rajabalipanah , A. Abdolali , Carbon 2019, 149, 125.

[22] B. Liu , Y. He , S. Wong , Y. Li , Adv. Opt. Mater. 2021, 9, 2001689.

[23] R. Feng , B. Ratni , J. Yi , K. Zhang , X. Ding , H. Zhang , A. de Lustrac , S. N. Burokur , Phys. Rev. Appl. 2020, 14, 014081.

[24] J. Sol , D. R. Smith , P. Del Hougne , Nat. Commun. 2022, 13, 1713. 35361758 10.1038/s41467-022-29354-w PMC8971527

[25] Z. Li , J. Liu , J. Zhang , L. Shao , C. Zhang , X. Wang , R. Jin , W. Zhu , Adv. Mater. Technol. 2022, 2200035.

[26] K. Chen , L. Cui , Y. Feng , J. Zhao , T. Jiang , B. Zhu , Opt. Express 2017, 25, 5571. 28380815 10.1364/OE.25.005571

[27] H.‐T. Chen , A. J. Taylor , N. Yu , Rep. Prog. Phys. 2016, 79, 076401. 27308726 10.1088/0034-4885/79/7/076401

[28] Y. Kim , G. Lee , J. Sung , J. Jang , B. Lee , Adv. Funct. Mater. 2022, 32, 2106050.

[29] S. Wang , P. C. Wu , V.‐C. Su , Y.‐C. Lai , M.‐K. Chen , H. Y. Kuo , B. H. Chen , Y. H. Chen , T.‐T. Huang , J.‐H. Wang , Nat. Nanotechnol. 2018, 13, 227. 29379204 10.1038/s41565-017-0052-4

[30] W. T. Chen , A. Y. Zhu , V. Sanjeev , M. Khorasaninejad , Z. Shi , E. Lee , F. Capasso , Nat. Nanotechnol. 2018, 13, 220. 29292382 10.1038/s41565-017-0034-6

[31] K. Chen , Y. Feng , F. Monticone , J. Zhao , B. Zhu , T. Jiang , L. Zhang , Y. Kim , X. Ding , S. Zhang , Adv. Mater. 2017, 29, 1606422. 10.1002/adma.201606422 28234431

[32] X. Chen , L. Huang , H. Mühlenbernd , G. Li , B. Bai , Q. Tan , G. Jin , C.‐W. Qiu , S. Zhang , T. Zentgraf , Nat. Commun. 2012, 3, 1198. 23149743 10.1038/ncomms2207 PMC3514495

[33] M. Khorasaninejad , W. Chen , A. Zhu , J. Oh , R. Devlin , D. Rousso , F. Capasso , Nano Lett. 2016, 16, 4595. 27267137 10.1021/acs.nanolett.6b01897

[34] M. Li , L. Shen , L. Jing , S. Xu , B. Zheng , X. Lin , Y. Yang , Z. Wang , H. Chen , Adv. Sci. 2019, 6, 1901434. 10.1002/advs.201901434 PMC6891917 31832314

[35] Q. Lou , C. Xue , Z. N. Chen , IEEE Trans. Antennas Propag. 2021, 69, 7394.

[36] Q. Lou , Z. N. Chen , IEEE Trans. Antennas Propag. 2022, 70, C3.

[37] Q. Lou , Z. N. Chen , IEEE Trans. Antennas Propag. 2021, 69, 6977. 10.1109/tap.2021.3083737 PMC9792069 36575724

[38] J. Soric , Y. Ra'di , D. Farfan , A. Alú , Nat. Commun. 2022, 13, 114. 35236850 10.1038/s41467-022-28714-w PMC8891352

[39] Y. Yang , L. Jing , B. Zheng , R. Hao , W. Yin , E. Li , C. M. Soukoulis , H. Chen , Adv. Mater. 2016, 28, 6866. 27218885 10.1002/adma.201600625

[40] X. Ni , Z. J. Wong , M. Mrejen , Y. Wang , X. Zhang , Science 2015, 349, 1310. 26383946 10.1126/science.aac9411

[41] G. Qu , W. Yang , Q. Song , Y. Liu , C.‐W. Qiu , J. Han , D.‐P. Tsai , S. Xiao , Nat. Commun. 2020, 11, 5484. 33127918 10.1038/s41467-020-19312-9 PMC7603497

[42] W. J. Kort‐Kamp , A. K. Azad , D. A. Dalvit , Phys. Rev. Lett. 2021, 127, 043603. 34355970 10.1103/PhysRevLett.127.043603

[43] G. D. Bai , T. J. Cui , Adv. Sci. 2020, 7, 2001648. 10.1002/advs.202001648 PMC7578880 33101865

[44] A. Arbabi , E. Arbabi , Y. Horie , S. M. Kamali , A. Faraon , Nat. Photonics 2017, 11, 415.

[45] W. Yang , K. Chen , Y. Zheng , W. Zhao , Q. Hu , K. Qu , T. Jiang , J. Zhao , Y. Feng , Adv. Sci. 2021, 8, 2100885. 10.1002/advs.202100885 PMC8564442 34486225

[46] Y. Saifullah , Q. Chen , G.‐M. Yang , A. B. Waqas , F. Xu , Opt. Express 2021, 29, 2658. 33726457 10.1364/OE.415730

[47] Q. Chen , Y. Saifullah , G.‐M. Yang , Y.‐Q. Jin , Opt. Express 2021, 29, 1470. 33726361 10.1364/OE.414572

[48] X. G. Zhang , W. X. Tang , W. X. Jiang , G. D. Bai , J. Tang , L. Bai , C. Qiu , T. J. Cui , Adv. Sci. 2018, 5, 1801028. 10.1002/advs.201801028 PMC6247069 30479931

[49] C. Wu , H. Yu , S. Lee , R. Peng , I. Takeuchi , M. Li , Nat. Commun. 2021, 12, 96. 33398011 10.1038/s41467-020-20365-z PMC7782756

[50] F. Ding , S. Zhong , S. I. Bozhevolnyi , Adv. Opt. Mater. 2018, 6, 1701204.

[51] S. Lepeshov , A. Krasnok , Nat. Nanotechnol. 2021, 16, 615. 33875870 10.1038/s41565-021-00892-6

[52] O. Buchnev , N. Podoliak , K. Kaltenecker , M. Walther , V. A. Fedotov , ACS Photonics 2020, 7, 3199.

[53] A. Komar , R. Paniagua‐Dominguez , A. Miroshnichenko , Y. F. Yu , Y. S. Kivshar , A. I. Kuznetsov , D. Neshev , ACS Photonics 2018, 5, 1742.

[54] Y. Liu , J. Song , W. Zhao , X. Ren , Q. Cheng , X. Luo , N. X. Fang , R. Hu , Nanophotonics 2020, 9, 855.

[55] A. Momeni , K. Rouhi , H. Rajabalipanah , A. Abdolali , Sci. Rep. 2018, 8, 6200. 29670151 10.1038/s41598-018-24553-2 PMC5906479

[56] S.‐F. Shi , B. Zeng , H.‐L. Han , X. Hong , H.‐Z. Tsai , H. Jung , A. Zettl , M. Crommie , F. Wang , Nano Lett. 2015, 15, 372. 25483819 10.1021/nl503670d

[57] Z. Miao , Q. Wu , X. Li , Q. He , K. Ding , Z. An , Y. Zhang , L. Zhou , Phys. Rev. X. 2015, 5, 041027.

[58] T. Kim , H. Kim , M. Kenney , H. S. Park , H. Kim , B. Min , S. Zhang , Adv. Opt. Mater. 2018, 6, 1700507.

[59] H. Liu , Z.‐H. Wang , L. Li , Y.‐X. Fan , Z.‐Y. Tao , Sci. Rep. 2019, 9, 5751. 30962484 10.1038/s41598-019-42293-9 PMC6453928

[60] J. A. Faber , A. F. Arrieta , A. R. Studart , Science 2018, 359, 1386. 29567709 10.1126/science.aap7753

[61] S. Li , H. Fang , S. Sadeghi , P. Bhovad , K. Wang , Adv. Mater. 2019, 31, 1805282. 10.1002/adma.201805282 30516852

[62] N. An , A. G. Domel , J. Zhou , A. Rafsanjani , K. Bertoldi , Adv. Funct. Mater. 2020, 30, 1906711.

[63] Y. Li , Q. Zhang , Y. Hong , J. Yin , Adv. Funct. Mater. 2021, 31, 2105641.

[64] Y. Tang , G. Lin , S. Yang , Y. K. Yi , R. D. Kamien , J. Yin , Adv. Mater. 2017, 29, 1604262. 10.1002/adma.201604262 28026066

[65] Y. Tang , Y. Li , Y. Hong , S. Yang , J. Yin , Proc. Natl. Acad. Sci. USA 2019, 116, 26407. 31843912 10.1073/pnas.1906435116 PMC6936366

[66] K. Bertoldi , V. Vitelli , J. Christensen , M. Van Hecke , Nat. Rev. Mater. 2017, 2, 17066.

[67] J. L. Silverberg , A. A. Evans , L. McLeod , R. C. Hayward , T. Hull , C. D. Santangelo , I. Cohen , Science 2014, 345, 647. 25104381 10.1126/science.1252876

[68] J. T. Overvelde , T. A. De Jong , Y. Shevchenko , S. A. Becerra , G. M. Whitesides , J. C. Weaver , C. Hoberman , K. Bertoldi , Nat. Commun. 2016, 7, 10929. 26965475 10.1038/ncomms10929 PMC4793042

[69] F. Liu , A. Pitilakis , M. S. Mirmoosa , O. Tsilipakos , X. Wang , A. C. Tasolamprou , S. Abadal , A. Cabellos‐Aparicio , E. Alarcón , C. Liaskos , in 2018 IEEE Int. Symposium on Circuits and Systems (ISCAS), IEEE, Piscataway, NJ 2018, pp. 1–5.

[70] X. Fu , F. Yang , C. Liu , X. Wu , T. J. Cui , Adv. Opt. Mater. 2020, 8, 1900628.

[71] Q. Ma , Q. R. Hong , G. D. Bai , H. B. Jing , R. Y. Wu , L. Bao , Q. Cheng , T. J. Cui , Phys. Rev. Appl. 2020, 13, 021003.

[72] M. Lin , M. Xu , X. Wan , H. Liu , Z. Wu , J. Liu , B. Deng , D. Guan , S. Zha , IEEE Internet Things J. 2021, 8, 10187.

[73] H. P. Wang , Y. X. Zhou , H. Li , G. D. Liu , S. M. Yin , P. J. Li , S. Y. Dong , C. Y. Gong , S. Y. Wang , Y. B. Li , Adv. Sci. 2022, 2105056.

[74] L. Li , H. Ruan , C. Liu , Y. Li , Y. Shuang , A. Alú , C.‐W. Qiu , T. J. Cui , Nat. Commun. 2019, 10, 1082. 30842417 10.1038/s41467-019-09103-2 PMC6403242

[75] L. Li , Y. Shuang , Q. Ma , H. Li , H. Zhao , M. Wei , C. Liu , C. Hao , C.‐W. Qiu , T. J. Cui , Light: Sci. Appl. 2019, 8, 97. 31645938 10.1038/s41377-019-0209-z PMC6804847

[76] L. Li , T. Jun Cui , W. Ji , S. Liu , J. Ding , X. Wan , Y. Bo Li , M. Jiang , C.‐W. Qiu , S. Zhang , Nat. Commun. 2017, 8, 197. 28775295 10.1038/s41467-017-00164-9 PMC5543116

[77] H. Yang , X. Cao , F. Yang , J. Gao , S. Xu , M. Li , X. Chen , Y. Zhao , Y. Zheng , S. Li , Sci. Rep. 2016, 6, 35692. 27774997 10.1038/srep35692 PMC5075904

[78] H. Zhang , N. Shlezinger , F. Guidi , D. Dardari , M. F. Imani , Y. C. Eldar , IEEE Trans. Wireless Commun. 2022, 21, 7476.

[79] A. H. Naqvi , S. Lim , IEEE Trans. Antennas Propag. 2019, 67, 3704.

[80] J. Wu , Z. Shen , S. Ge , B. Chen , Z. Shen , T. Wang , C. Zhang , W. Hu , K. Fan , W. Padilla , Appl. Phys. Lett. 2020, 116, 131104.

[81] S. E. Hosseininejad , K. Rouhi , M. Neshat , A. Cabellos‐Aparicio , S. Abadal , E. Alarcón , IEEE Trans. Nanotechnol. 2019, 18, 734.

[82] X. Fu , L. Shi , J. Yang , Y. Fu , C. Liu , J. W. Wu , F. Yang , L. Bao , T. J. Cui , ACS Appl. Mater. Interfaces 2022, 14, 22287. 35476394 10.1021/acsami.2c02601

[83] C. X. Liu , F. Yang , X. J. Fu , J. W. Wu , L. Zhang , J. Yang , T. J. Cui , Adv. Opt. Mater. 2021, 9, 2100932.

[84] S. Taravati , G. V. Eleftheriades , Nat. Commun. 2021, 12, 4414. 34285230 10.1038/s41467-021-24749-7 PMC8292412

[85] G. K. Shirmanesh , R. Sokhoyan , P. C. Wu , H. A. Atwater , ACS Nano 2020, 14, 6912. 32352740 10.1021/acsnano.0c01269

[86] W. Tang , X. Li , J. Y. Dai , S. Jin , Y. Zeng , Q. Cheng , T. J. Cui , China Commun. 2019, 16, 46.

[87] W. Tang , M. Z. Chen , J. Y. Dai , Y. Zeng , X. Zhao , S. Jin , Q. Cheng , T. J. Cui , IEEE Wireless Commun. 2020, 27, 180.

[88] J. Y. Dai , W. Tang , M. Z. Chen , C. H. Chan , Q. Cheng , S. Jin , T. J. Cui , IEEE Trans. Microwave Theory Tech. 2021, 69, 1493.

[89] J. Zhao , X. Yang , J. Y. Dai , Q. Cheng , X. Li , N. H. Qi , J. C. Ke , G. D. Bai , S. Liu , S. Jin , Natl. Sci. Rev. 2019, 6, 231. 34691861 10.1093/nsr/nwy135 PMC8291514

[90] X. Wan , C. K. Xiao , H. Huang , Q. Xiao , W. Xu , J. W. Wang , Z. A. Huang , Q. Cheng , S. Jin , T. J. Cui , Adv. Mater. Technol. 2021, 6, 2001254.

[91] H. Zhao , Y. Shuang , M. Wei , T. J. Cui , P. d. Hougne , L. Li , Nat. Commun. 2020, 11, 3926. 32764638 10.1038/s41467-020-17808-y PMC7413398

[92] X. Wan , C. Xiao , H. Huang , Q. Xiao , W. Xu , Y. Li , J. Eisenbeis , J. Wang , Z. Huang , Q. Cheng , Engineering 2022, 8, 86.

[93] T. J. Cui , S. Liu , G. D. Bai , Q. Ma , Research 2019, 2019, 2584509. 31549052 10.34133/2019/2584509 PMC6750087

[94] L. Zhang , M. Z. Chen , W. Tang , J. Y. Dai , L. Miao , X. Y. Zhou , S. Jin , Q. Cheng , T. J. Cui , Nat. Electron. 2021, 4, 218.

[95] X. G. Zhang , Y. L. Sun , B. Zhu , W. X. Jiang , Q. Yu , H. W. Tian , C.‐W. Qiu , Z. Zhang , T. J. Cui , Light: Sci. Appl. 2022, 11, 126. 35513383 10.1038/s41377-022-00817-5 PMC9072331

[96] J. Guo , G. Xu , D. Tian , Z. Qu , C. Qiu , Adv. Mater. 2022, 34, 2201093. 10.1002/adma.202201093 35415933

[97] C. Qian , B. Zheng , Y. Shen , L. Jing , E. Li , L. Shen , H. Chen , Nat. Photonics 2020, 14, 383.

[98] Q. Ma , G. D. Bai , H. B. Jing , C. Yang , L. Li , T. J. Cui , Light: Sci. Appl. 2019, 8, 98. 31700618 10.1038/s41377-019-0205-3 PMC6823478

[99] Y. She , C. Ji , C. Huang , Z. Zhang , J. Liao , J. Wang , X. Luo , Photonics Res. 2022, 10, 769.

[100] Q. Ma , Q. R. Hong , X. X. Gao , H. B. Jing , C. Liu , G. D. Bai , Q. Cheng , T. J. Cui , Nanophotonics 2020, 9, 3271.

[101] A. E. Cardin , S. R. Silva , S. R. Vardeny , W. J. Padilla , A. Saxena , A. J. Taylor , W. J. Kort‐Kamp , H.‐T. Chen , D. A. Dalvit , A. K. Azad , Nat. Commun. 2020, 11, 1469. 32193393 10.1038/s41467-020-15273-1 PMC7081213

[102] A. M. Shaltout , V. M. Shalaev , M. L. Brongersma , Science 2019, 364, eaat3100. 31097638 10.1126/science.aat3100

[103] Y. Hu , M. Tong , Z. Xu , X. Cheng , T. Jiang , Small 2021, 17, 2006489. 10.1002/smll.202006489 33838009

[104] L. Cong , R. Singh , Adv. Mater. 2020, 32, 2001418. 10.1002/adma.202001418 32468602

[105] E. Mikheeva , C. Kyrou , F. Bentata , S. Khadir , S. Cueff , P. Genevet , ACS Photonics 2022, 9, 1458.

[106] H. Wu , X. X. Gao , L. Zhang , G. D. Bai , Q. Cheng , L. Li , T. J. Cui , Light: Sci. Appl. 2020, 9, 198. 33318469 10.1038/s41377-020-00441-1 PMC7736919

[107] H. Rajabalipanah , A. Abdolali , K. Rouhi , IEEE J. Emerg. Sel. Top. Circuits Syst. 2020, 10, 75.

[108] Z. Chen , Y. Peng , H. Li , J. Liu , Y. Ding , B. Liang , X.‐F. Zhu , Y. Lu , J. Cheng , A. Alú , Sci. Adv. 2021, 7, eabj1198. 34731003 10.1126/sciadv.abj1198 PMC8565901

[109] D. L. Sounas , C. Caloz , A. Alù , Nat. Commun. 2013, 4, 2407. 23994940 10.1038/ncomms3407

[110] N. A. Estep , D. L. Sounas , J. Soric , A. Alu , Nat. Phys. 2014, 10, 923.

[111] Y. Hadad , J. C. Soric , A. Alu , Proc. Natl. Acad. Sci. USA 2016, 113, 3471. 26984502 10.1073/pnas.1517363113 PMC4822645

[112] D. L. Sounas , A. Alu , Nat. Photonics 2017, 11, 774.

[113] C. Zhang , J. Yang , L. X. Yang , J. C. Ke , M. Z. Chen , W. K. Cao , M. Chen , Z. H. Wu , J. F. Chen , Q. Cheng , Nanophotonics 2020, 9, 2771.

[114] L. Zhang , T. J. Cui , Research 2021, 2021, 9802673. 34386772 10.34133/2021/9802673 PMC8328401

[115] L. Zhang , J. Y. Dai , M. Moccia , G. Castaldi , T. J. Cui , V. Galdi , EPJ Appl. Metamater. 2020, 7, 7.

[116] X. Wang , J. Han , S. Tian , D. Xia , L. Li , T. J. Cui , Adv. Sci. 2022, 9, 2105960. 10.1002/advs.202105960 PMC9008792 35142443

[117] M. Z. Chen , W. Tang , J. Y. Dai , J. C. Ke , L. Zhang , C. Zhang , J. Yang , L. Li , Q. Cheng , S. Jin , Natl. Sci. Rev. 2022, 9, nwab134. 35079409 10.1093/nsr/nwab134 PMC8783670

[118] M. M. Salary , S. Jafar‐Zanjani , H. Mosallaei , New J. Phys. 2018, 20, 123023.

[119] M. M. Salary , S. Farazi , H. Mosallaei , Adv. Opt. Mater. 2019, 7, 1900843.

[120] D. Ramaccia , D. L. Sounas , A. Alú , A. Toscano , F. Bilotti , Phys. Rev. B 2017, 95, 075113.

[121] B. Liu , H. Giddens , Y. Li , Y. He , S.‐W. Wong , Y. Hao , Opt. Express 2020, 28, 3745. 32122036 10.1364/OE.382700

[122] B. Liu , Y. He , S.‐W. Wong , Y. Li , Opt. Express 2021, 29, 740. 33726304 10.1364/OE.414408

[123] D. Ramaccia , D. L. Sounas , A. Alu , A. Toscano , F. Bilotti , IEEE Trans. Antennas Propag. 2019, 68, 1607.

[124] Z. Liu , Z. Li , K. Aydin , ACS Photonics 2016, 3, 2035.

[125] K. Lee , J. Son , J. Park , B. Kang , W. Jeon , F. Rotermund , B. Min , Nat. Photonics 2018, 12, 765.

[126] Z. Wu , A. Grbic , IEEE Trans. Antennas Propag. 2019, 68, 1599.

[127] M. Saikia , K. V. Srivastava , S. A. Ramakrishna , IEEE Trans. Antennas Propag. 2019, 68, 2937.

[128] S. Basharat , S. A. Hassan , H. Pervaiz , A. Mahmood , Z. Ding , M. Gidlund , IEEE Wireless Commun. 2021, 28, 184.

[129] X. Lei , M. Wu , F. Zhou , X. Tang , R. Q. Hu , P. Fan , IEEE Wireless Commun. 2021, 28, 210.

[130] Y. Wang , W. Zhang , Y. Chen , C.‐X. Wang , J. Sun , IEEE Commun. Lett. 2022, 26, 1413.

[131] H. Zhang , B. Di , L. Song , Z. Han , Reconfigurable Intelligent Surface‐Empowered 6G, Springer, Cham 2021.

[132] L.‐C. Wang , H. Peng , A. C.‐S. Huang , A.‐H. Tsai , in 2021 30th Wireless and Optical Communications Conf. (WOCC), IEEE, Piscataway, NJ 2021, pp. 295–297.

[133] Y. Liu , X. Liu , X. Mu , T. Hou , J. Xu , M. Di Renzo , N. Al‐Dhahir , IEEE Commun. Surv. Tut. 2021, 23, 1546.

[134] C. Pan , H. Ren , K. Wang , J. F. Kolb , M. Elkashlan , M. Chen , M. Di Renzo , Y. Hao , J. Wang , A. L. Swindlehurst , IEEE Commun. Mag. 2021, 59, 14.

[135] X. Tan , Z. Sun , D. Koutsonikolas , J. M. Jornet , in IEEE INFOCOM 2018‐IEEE Conf. on Computer Communications, IEEE, Piscataway, NJ 2018, pp. 270–278.

[136] X. Tan , Z. Sun , J. M. Jornet , D. Pados , in 2016 IEEE Int. Conf. on Communications (ICC), IEEE, Piscataway, NJ 2016, pp. 1–6.

[137] z. Özdogan , E. Björnson , E. G. Larsson , IEEE Wireless Commun. Lett. 2019, 9, 581.

[138] X. Yu , D. Xu , Y. Sun , D. W. K. Ng , R. Schober , IEEE J. Sel. Areas Commun. 2020, 38, 2637.

[139] C. Pan , H. Ren , K. Wang , W. Xu , M. Elkashlan , A. Nallanathan , L. Hanzo , IEEE Trans. Wireless Commun. 2020, 19, 5218.

[140] M. Cui , G. Zhang , R. Zhang , IEEE Wireless Commun. Lett. 2019, 8, 1410.

[141] Q. Wu , R. Zhang , IEEE Commun. Mag. 2019, 58, 106.

[142] B. Zheng , R. Zhang , IEEE Wireless Commun. Lett. 2019, 9, 518.

[143] S. Zhang , R. Zhang , IEEE J. Sel. Areas Commun. 2020, 38, 1823.

[144] Q. Wu , R. Zhang , IEEE Trans. Wireless Commun. 2019, 18, 5394.

[145] Y.‐C. Liang , R. Long , Q. Zhang , J. Chen , H. V. Cheng , H. Guo , J. Commun. Network 2019, 4, 40.

[146] S. Hu , F. Rusek , O. Edfors , IEEE Trans. Signal Process. 2018, 66, 2746.

[147] D. Dardari , IEEE J. Sel. Areas Commun. 2020, 38, 2526.

[148] S. Hu , F. Rusek , O. Edfors , IEEE Trans. Signal Process. 2018, 66, 1761.

[149] C. Huang , G. C. Alexandropoulos , A. Zappone , M. Debbah , C. Yuen , in 2018 IEEE Globecom Workshops (GC Wkshps), IEEE, Piscataway, NJ 2018, pp. 1–6.

[150] C. Huang , A. Zappone , M. Debbah , C. Yuen , in 2018 IEEE Int. Conf. on Acoustics, Speech and Signal Processing (ICASSP), IEEE, Piscataway, NJ 2018, pp. 3714–3718.

[151] M. Di Renzo , A. Zappone , M. Debbah , M.‐S. Alouini , C. Yuen , J. De Rosny , S. Tretyakov , IEEE J. Sel. Areas Commun. 2020, 38, 2450.

[152] M. D. Renzo , M. Debbah , D.‐T. Phan‐Huy , A. Zappone , M.‐S. Alouini , C. Yuen , V. Sciancalepore , G. C. Alexandropoulos , J. Hoydis , H. Gacanin , Eurasip J. Wireless Commun. Network 2019, 2019, 955.

[153] E. C. Strinati , G. C. Alexandropoulos , H. Wymeersch , B. Denis , V. Sciancalepore , R. D'Errico , A. Clemente , D.‐T. Phan‐Huy , E. De Carvalho , P. Popovski , IEEE Commun. Mag. 2021, 59, 99.

[154] R. Liu , Q. Wu , M. Di Renzo , Y. Yuan , IEEE Wireless Commun. 2022, 29, 202.

[155] A. Zappone , M. Di Renzo , F. Shams , X. Qian , M. Debbah , IEEE Trans. Wireless Commun. 2020, 20, 126.

[156] X. Gu , W. Duan , G. Zhang , Y. Ji , M. Wen , P.‐H. Ho , IEEE Trans. Veh. Technol. 2022.

[157] Y. Ai , A. Felipe , L. Kong , M. Cheffena , S. Chatzinotas , B. Ottersten , IEEE Trans. Veh. Technol. 2021, 70, 7272.

[158] Y. Zhu , B. Mao , Y. Kawamoto , N. Kato , IEEE Veh. Technol. Mag. 2021, 16, 48.

[159] K. Yang , Y. Shi , Y. Zhou , Z. Yang , L. Fu , W. Chen , IEEE Network 2020, 34, 16.

[160] S. Dinh‐Van , T. M. Hoang , R. Trestian , H. X. Nguyen , IEEE Internet Things J. 2022.

[161] Y. Zeng , R. Zhang , T. J. Lim , IEEE Commun. Mag. 2016, 54, 36.

[162] M. A. ElMossallamy , H. Zhang , L. Song , K. G. Seddik , Z. Han , G. Y. Li , IEEE Trans. Cogn. Commun. 2020, 6, 990.

[163] S. Li , B. Duo , M. Di Renzo , M. Tao , X. Yuan , IEEE Trans. Wirel. Commun. 2021, 20, 6402.

[164] C. Huang , A. Zappone , G. C. Alexandropoulos , M. Debbah , C. Yuen , IEEE Trans. Wirel. Commun. 2019, 18, 4157.

[165] X. Yuan , Y.‐J. A. Zhang , Y. Shi , W. Yan , H. Liu , IEEE Wirel. Commun. 2021, 28, 136.

[166] W. Tang , M. Z. Chen , X. Chen , J. Y. Dai , Y. Han , M. Di Renzo , Y. Zeng , S. Jin , Q. Cheng , T. J. Cui , IEEE Trans. Wireless Commun. 2020, 20, 421.

[167] S. Li , B. Duo , X. Yuan , Y.‐C. Liang , M. Di Renzo , IEEE Wireless Commun. Lett. 2020, 9, 716.

[168] Q. Wu , X. Guan , R. Zhang , Proc. IEEE 2021, 110, 150.

[169] Q. Wu , R. Zhang , IEEE Wireless Commun. Lett. 2019, 9, 586.

[170] Q. Wu , R. Zhang , IEEE J. Sel. Areas Commun. 2020, 38, 1735. PMC7537467 33029039

[171] J. Xu , Y. Liu , X. Mu , O. A. Dobre , IEEE Commun. Lett. 2021, 25, 3134.

[172] H. Niu , Z. Chu , F. Zhou , Z. Zhu , IEEE Commun. Lett. 2021, 25, 3498.

[173] X. Mu , Y. Liu , L. Guo , J. Lin , R. Schober , IEEE Trans. Wireless Commun. 2021.

[174] Q. He , S. Sun , L. Zhou , Research 2019, 2019, 1849272. 31549047 10.34133/2019/1849272 PMC6750114

[175] O. Tsilipakos , A. C. Tasolamprou , A. Pitilakis , F. Liu , X. Wang , M. S. Mirmoosa , D. C. Tzarouchis , S. Abadal , H. Taghvaee , C. Liaskos , Adv. Opt. Mater. 2020, 8, 2000783.

[176] T. Cui , B. Bai , H. Sun , Adv. Funct. Mater. 2019, 29, 1806692.

[177] L. Kang , R. P. Jenkins , D. H. Werner , Adv. Opt. Mater. 2019, 7, 1801813.

[178] F. Ding , Y. Yang , S. I. Bozhevolnyi , Adv. Opt. Mater. 2019, 7, 1801709.

[179] J. Qi , Z. Chen , P. Jiang , W. Hu , Y. Wang , Z. Zhao , X. Cao , S. Zhang , R. Tao , Y. Li , Adv. Sci. 2022, 2102662. 10.1002/advs.202102662 PMC8728820 34716676

[180] L. Bao , T. J. Cui , Microw. Opt. Technol. Lett. 2020, 62, 9.

[181] S. Gong , X. Lu , D. T. Hoang , D. Niyato , L. Shu , D. I. Kim , Y.‐C. Liang , IEEE Commun. Surv. Tut. 2020, 22, 2283.

[182] Q. Wu , S. Zhang , B. Zheng , C. You , R. Zhang , IEEE Trans. Commun. 2021, 69, 3313.

[183] A. C. Tasolamprou , A. D. Koulouklidis , C. Daskalaki , C. P. Mavidis , G. Kenanakis , G. Deligeorgis , Z. Viskadourakis , P. Kuzhir , S. Tzortzakis , M. Kafesaki , ACS Photonics 2019, 6, 720. 30918912 10.1021/acsphotonics.8b01595 PMC6429433

[184] Y. Hu , T. Jiang , J. Zhou , H. Hao , H. Sun , H. Ouyang , M. Tong , Y. Tang , H. Li , J. You , Adv. Opt. Mater. 2019, 7, 1901050.

[185] J. Park , J.‐H. Kang , S. J. Kim , X. Liu , M. L. Brongersma , Nano Lett. 2017, 17, 407. 27936784 10.1021/acs.nanolett.6b04378

[186] X. G. Zhang , Q. Yu , W. X. Jiang , Y. L. Sun , L. Bai , Q. Wang , C. Qiu , T. J. Cui , Adv. Sci. 2020, 7, 1903382. 10.1002/advs.201903382 PMC7284210 32537403

[187] H.‐S. Ee , R. Agarwal , Nano Lett. 2016, 16, 2818. 26986191 10.1021/acs.nanolett.6b00618

[188] Y. Wang , L. Li , D. Hofmann , J. E. Andrade , C. Daraio , Nature 2021, 596, 238. 34381233 10.1038/s41586-021-03698-7

[189] Y. Chen , X. Duan , M. Matuschek , Y. Zhou , F. Neubrech , H. Duan , N. Liu , Nano Lett. 2017, 17, 5555. 28721735 10.1021/acs.nanolett.7b02336

[190] J. Li , S. Kamin , G. Zheng , F. Neubrech , S. Zhang , N. Liu , Sci. Adv. 2018, 4, eaar6768. 29922715 10.1126/sciadv.aar6768 PMC6003725

[191] Y. Wang , P. Landreman , D. Schoen , K. Okabe , A. Marshall , U. Celano , H.‐S. P. Wong , J. Park , M. L. Brongersma , Nat. Nanotechnol. 2021, 16, 667. 33875869 10.1038/s41565-021-00882-8

[192] Z. Zhu , P. G. Evans , R. F. Haglund Jr , J. G. Valentine , Nano Lett. 2017, 17, 4881. 28731722 10.1021/acs.nanolett.7b01767

[193] M. Y. Shalaginov , S. An , Y. Zhang , F. Yang , P. Su , V. Liberman , J. B. Chou , C. M. Roberts , M. Kang , C. Rios , Nat. Commun. 2021, 12, 1225. 33619270 10.1038/s41467-021-21440-9 PMC7900249

[194] C. Meng , S. Tang , F. Ding , S. I. Bozhevolnyi , ACS Photonics 2020, 7, 1849.

[195] E. Almeida , O. Bitton , Y. Prior , Nat. Commun. 2016, 7, 12533. 27545581 10.1038/ncomms12533 PMC4996937

[196] M. L. Tseng , Y. Jahani , A. Leitis , H. Altug , ACS Photonics 2020, 8, 47.

[197] J. Y. Dai , J. Zhao , Q. Cheng , T. J. Cui , Light: Sci. Appl. 2018, 7, 90. 30479756 10.1038/s41377-018-0092-z PMC6249241

[198] E. Arbabi , S. M. Kamali , A. Arbabi , A. Faraon , ACS Photonics 2018, 5, 3132.

[199] S. Abdollahramezani , O. Hemmatyar , M. Taghinejad , H. Taghinejad , A. Krasnok , A. A. Eftekhar , C. Teichrib , S. Deshmukh , M. A. El‐Sayed , E. Pop , M. Wuttig , A. Alú , W. Cai , Ali Adibi , Nat. Commun. 2022, 13, 1696. 35354813 10.1038/s41467-022-29374-6 PMC8967895

[200] A. Tripathi , J. John , S. Kruk , Z. Zhang , H. S. Nguyen , L. Berguiga , P. R. Romeo , R. Orobtchouk , S. Ramanathan , Y. Kivshar , ACS Photonics 2021, 8, 1206.

[201] J. Ge , Y. Zhang , H. Dong , L. Zhang , ACS Appl. Nano Mater. 2022, 5, 5569.

[202] K. Sun , C. A. Riedel , A. Urbani , M. Simeoni , S. Mengali , M. Zalkovskij , B. Bilenberg , C. De Groot , O. L. Muskens , ACS Photonics 2018, 5, 2280.

[203] J. Shabanpour , J. Mater. Chem. C 2020, 8, 7189.

[204] M. R. Shcherbakov , S. Liu , V. V. Zubyuk , A. Vaskin , P. P. Vabishchevich , G. Keeler , T. Pertsch , T. V. Dolgova , I. Staude , I. Brener , Nat. Commun. 2017, 8, 17. 28500308 10.1038/s41467-017-00019-3 PMC5432034

[205] M. Taghinejad , H. Taghinejad , Z. Xu , Y. Liu , S. P. Rodrigues , K. Lee , T. Lian , A. Adibi , W. Cai , Adv. Mater. 2018, 30, 1704915. 10.1002/adma.201704915 29333735

[206] M. Taghinejad , H. Taghinejad , Z. Xu , K.‐T. Lee , S. P. Rodrigues , J. Yan , A. Adibi , T. Lian , W. Cai , Nano Lett. 2018, 18, 5544. 30071164 10.1021/acs.nanolett.8b01946

[207] W. J. Padilla , A. J. Taylor , C. Highstrete , M. Lee , R. D. Averitt , Phys. Rev. Lett. 2006, 96, 107401. 16605787 10.1103/PhysRevLett.96.107401

[208] I. Chatzakis , L. Luo , J. Wang , N.‐H. Shen , T. Koschny , J. Zhou , C. Soukoulis , Phys. Rev. B 2012, 86, 125110.

[209] D. Roy Chowdhury , R. Singh , J. F. O'Hara , H.‐T. Chen , A. J. Taylor , A. K. Azad , Appl. Phys. Lett. 2011, 99, 231101.

[210] Y. Fan , N.‐H. Shen , F. Zhang , Q. Zhao , Z. Wei , P. Zhang , J. Dong , Q. Fu , H. Li , C. M. Soukoulis , ACS Photonics 2018, 5, 1612.

[211] P.‐Y. Chen , J. Jung , Phys. Rev. Appl. 2016, 5, 064018.

[212] C. M. Watts , A. Pedross‐Engel , D. R. Smith , M. S. Reynolds , J. Opt. Soc. Am. B 2017, 34, 300. 10.1364/JOSAA.34.000A22 28463331

[213] Q. Wang , X. G. Zhang , H. W. Tian , W. X. Jiang , D. Bao , H. L. Jiang , Z. J. Luo , L. T. Wu , T. J. Cui , Adv. Theory Simul. 2019, 2, 1900141.

[214] A. Komar , Z. Fang , J. Bohn , J. Sautter , M. Decker , A. Miroshnichenko , T. Pertsch , I. Brener , Y. S. Kivshar , I. Staude , Appl. Phys. Lett. 2017, 110, 071109.

[215] E. Feigenbaum , K. Diest , H. A. Atwater , Nano Lett. 2010, 10, 2111. 20481480 10.1021/nl1006307

[216] K. Thyagarajan , R. Sokhoyan , L. Zornberg , H. A. Atwater , Adv. Mater. 2017, 29, 1701044. 10.1002/adma.201701044 28612946

[217] J. Zhang , Z. Li , L. Shao , W. Zhu , Carbon 2021, 176, 374.

[218] Y. Fan , N.‐H. Shen , T. Koschny , C. M. Soukoulis , ACS Photonics 2015, 2, 151.

[219] Y. Wang , T. Li , S. Zhu , Opt. Lett. 2017, 42, 2247. 28614323 10.1364/OL.42.002247

[220] J. Han , L. Li , X. Ma , X. Gao , Y. Mu , G. Liao , Z. J. Luo , T. J. Cui , IEEE Trans. Ind. Electron. 2021, 69, 8524.

[221] X. Wan , Q. Xiao , Y. Z. Zhang , Y. Li , J. Eisenbeis , J. W. Wang , Z. A. Huang , H. X. Liu , T. Zwick , T. J. Cui , IEEE Antennas Wireless Propag. Lett. 2021, 20, 381.

[222] J. C. Liang , Q. Cheng , Y. Gao , C. Xiao , S. Gao , L. Zhang , S. Jin , T. J. Cui , IEEE Trans. Antennas Propag. 2021, 10.1109/TAP.2021.3130108.

[223] T. Badloe , J. Lee , J. Seong , J. Rho , Adv. Photonics Res. 2021, 2, 2000205.

[224] W. Wu , W. Hu , G. Qian , H. Liao , X. Xu , F. Berto , Mater. Des. 2019, 180, 107950.

[225] X. Yu , J. Zhou , H. Liang , Z. Jiang , L. Wu , Prog. Mater. Sci. 2018, 94, 114.

[226] F. Zangeneh‐Nejad , R. Fleury , Rev. Phys. 2019, 4, 100031.

[227] M. Kadic , G. W. Milton , M. van Hecke , M. Wegener , Nat. Rev. Phys. 2019, 1, 198.

[228] G. Ma , P. Sheng , Sci. Adv. 2016, 2, e1501595. 26933692 10.1126/sciadv.1501595 PMC4771441

[229] J. Qi , Z. Chen , P. Jiang , W. Hu , Y. Wang , Z. Zhao , X. Cao , S. Zhang , R. Tao , Y. Li , D. Fang , Adv. Sci. 2022, 9, 2102662. 10.1002/advs.202102662 PMC8728820 34716676

[230] Y. Chang , J. Wei , C. Lee , Nanophotonics 2020, 9, 3049.

[231] X. Zhao , G. Duan , A. Li , C. Chen , X. Zhang , Microsyst. Nanoeng. 2019, 5, 5. 31057932 10.1038/s41378-018-0042-1 PMC6348284

[232] Z. Ren , Y. Chang , Y. Ma , K. Shih , B. Dong , C. Lee , Adv. Opt. Mater. 2020, 8, 1900653.

[233] Y. Hui , J. S. Gomez‐Diaz , Z. Qian , A. Alu , M. Rinaldi , Nat. Commun. 2016, 7, 11249. 27080018 10.1038/ncomms11249 PMC4835539

[234] C. P. Ho , P. Pitchappa , Y.‐S. Lin , C.‐Y. Huang , P. Kropelnicki , C. Lee , Appl. Phys. Lett. 2014, 104, 161104.

[235] A. X. Lalas , N. V. Kantartzis , T. D. Tsiboukis , Microsyst. Technol. 2015, 21, 2097.

[236] X. Li , T. Yang , W. Zhu , X. Li , Microsyst. Technol. 2013, 19, 1145.

[237] Y. H. Fu , A. Q. Liu , W. M. Zhu , X. M. Zhang , D. P. Tsai , J. B. Zhang , T. Mei , J. F. Tao , H. C. Guo , X. H. Zhang , Adv. Funct. Mater. 2011, 21, 3589.

[238] W. M. Zhu , A. Q. Liu , X. M. Zhang , D. P. Tsai , T. Bourouina , J. H. Teng , X. H. Zhang , H. C. Guo , H. Tanoto , T. Mei , Adv. Mater. 2011, 23, 1792. 21491512 10.1002/adma.201004341

[239] W. Zhu , A. Liu , T. Bourouina , D. P. Tsai , J. Teng , X. Zhang , G. Lo , D. Kwong , N. Zheludev , Nat. Commun. 2012, 3, 1274. 23232404 10.1038/ncomms2285 PMC3535344

[240] K. Shih , P. Pitchappa , M. Manjappa , C. P. Ho , R. Singh , B. Yang , N. Singh , C. Lee , Appl. Phys. Lett. 2017, 110, 161108.

[241] S. Sun , W. Yang , C. Zhang , J. Jing , Y. Gao , X. Yu , Q. Song , S. Xiao , ACS Nano 2018, 12, 2151. 29469563 10.1021/acsnano.7b07121

[242] T. H. Le , T. Tanaka , ACS Nano 2017, 11, 9780. 28945355 10.1021/acsnano.7b02743

[243] D. Rodrigo , A. Tittl , N. Ait‐Bouziad , A. John‐Herpin , O. Limaj , C. Kelly , D. Yoo , N. J. Wittenberg , S.‐H. Oh , H. A. Lashuel , Nat. Commun. 2018, 9, 2160. 29867181 10.1038/s41467-018-04594-x PMC5986821

[244] J. Lee , S. Jung , P. Chen , F. Lu , F. Demmerle , G. Boehm , M. Amann , A. Alú , M. A. Belkin , Adv. Opt. Mater. 2014, 2, 1057.

[245] X. Yin , T. Steinle , L. Huang , T. Taubner , M. Wuttig , T. Zentgraf , H. Giessen , Light: Sci. Appl. 2017, 6, e17016. 30167272 10.1038/lsa.2017.16 PMC6062225

[246] Y. Zheng , K. Chen , W. Yang , L. Wu , K. Qu , J. Zhao , T. Jiang , Y. Feng , Adv. Funct. Mater. 2022, 32, 2107699.

[247] H.‐X. Xu , M. Wang , G. Hu , S. Wang , Y. Wang , C. Wang , Y. Zeng , J. Li , S. Zhang , W. Huang , Research 2021, 2021.

[248] S. M. Kamali , A. Arbabi , E. Arbabi , Y. Horie , A. Faraon , Nat. Commun. 2016, 7, 11618. 27193141 10.1038/ncomms11618 PMC4874029

[249] P. Gutruf , C. Zou , W. Withayachumnankul , M. Bhaskaran , S. Sriram , C. Fumeaux , ACS Nano 2016, 10, 133. 26617198 10.1021/acsnano.5b05954

[250] S. Babaee , J. T. Overvelde , E. R. Chen , V. Tournat , K. Bertoldi , Sci. Adv. 2016, 2, e1601019. 28138527 10.1126/sciadv.1601019 PMC5262461

[251] R. Kaissner , J. Li , W. Lu , X. Li , F. Neubrech , J. Wang , N. Liu , Sci. Adv. 2021, 7, eabd9450. 33952513 10.1126/sciadv.abd9450 PMC8099187

[252] J. Peng , H.‐H. Jeong , Q. Lin , S. Cormier , H.‐L. Liang , M. F. De Volder , S. Vignolini , J. J. Baumberg , Sci. Adv. 2019, 5, eaaw2205. 31093530 10.1126/sciadv.aaw2205 PMC6510554

[253] Y. Li , J. van de Groep , A. A. Talin , M. L. Brongersma , Nano Lett. 2019, 19, 7988. 31560552 10.1021/acs.nanolett.9b03143

[254] S. Zanotto , A. Blancato , A. Buchheit , M. Muñoz–Castro , H. Wiemhöfer , F. Morichetti , A. Melloni , Adv. Opt. Mater. 2017, 5, 1600732.

[255] P. Yu , J. Li , X. Li , G. Schütz , M. Hirscher , S. Zhang , N. Liu , ACS Nano 2019, 13, 7100. 31083965 10.1021/acsnano.9b02425 PMC6595502

[256] S. Chen , E. S. Kang , M. Shiran Chaharsoughi , V. Stanishev , P. Kühne , H. Sun , C. Wang , M. Fahlman , S. Fabiano , V. Darakchieva , Nat. Nanotechnol. 2020, 15, 35. 31819242 10.1038/s41565-019-0583-y

[257] H. Yang , F. Yang , X. Cao , S. Xu , J. Gao , X. Chen , M. Li , T. Li , IEEE Trans. Antennas Propag. 2017, 65, 3024.

[258] L. Li , T. J. Cui , W. Ji , S. Liu , J. Ding , X. Wan , Y. B. Li , M. Jiang , C.‐W. Qiu , S. Zhang , Nat. Commun. 2017, 8, 197. 28775295 10.1038/s41467-017-00164-9 PMC5543116

[259] R. Feng , B. Ratni , J. Yi , Z. Jiang , H. Zhang , A. de Lustrac , S. N. Burokur , Adv. Opt. Mater. 2020, 8, 2001084.

[260] Y. Saifullah , G. Yang , F. Xu , in 2021 Cross Strait Radio Science and Wireless Technology Conference (CSRSWTC), IEEE, Piscataway, NJ 2021, pp. 89–91.

[261] S. Li , F. Xu , X. Wan , T. J. Cui , Y.‐Q. Jin , IEEE Trans. Antennas Propag. 2020, 69, 2958.

[262] A. Pedross‐Engel , C. M. Watts , D. R. Smith , M. S. Reynolds , IEEE Trans. Geosci. Remote Sens. 2017, 55, 3764.

[263] X. Wan , T. Y. Chen , X. Q. Chen , L. Zhang , T. J. Cui , IEEE Trans. Antennas Propag. 2018, 66, 4942.

[264] T. Sleasman , M. Boyarsky , L. Pulido‐Mancera , T. Fromenteze , M. F. Imani , M. S. Reynolds , D. R. Smith , IEEE Trans. Antennas Propag. 2017, 65, 6864. 10.1364/JOSAA.34.000A22 28463331

[265] S. Li , Z. Liu , S. Fu , Y. Wang , F. Xu , IEEE Trans. Antennas Propag. 2022, 69, 2958.

[266] H. L. Wang , H. F. Ma , M. Chen , S. Sun , T. J. Cui , Adv. Funct. Mater. 2021, 31, 2100275.

[267] B. D. Nguyen , C. Pichot , IEEE Antennas Wireless Propag. Lett. 2018, 18, 98.

[268] A. Clemente , L. Dussopt , R. Sauleau , P. Potier , P. Pouliguen , IEEE Trans. Antennas Propag. 2012, 60, 2260.

[269] J. Y. Lau , S. V. Hum , IEEE Trans. Microwave Theory Tech. 2010, 58, 3547.

[270] C.‐W. Luo , G. Zhao , Y.‐C. Jiao , G.‐T. Chen , Y.‐D. Yan , IEEE Antennas Wireless Propag. Lett. 2021, 20, 798.

[271] Y. Xiao , B. Xi , M. Xiang , F. Yang , Z. Chen , IEEE Antennas Wireless Propag. Lett. 2021, 20, 1908.

[272] J. Y. Lau , S. V. Hum , IEEE Trans. Antennas Propag. 2010, 59, 70.

[273] L. Di Palma , A. Clemente , L. Dussopt , R. Sauleau , P. Potier , P. Pouliguen , IEEE Trans. Antennas Propag. 2016, 65, 529.

[274] M. Wang , S. Xu , F. Yang , M. Li , IEEE Trans. Antennas Propag. 2019, 67, 3500.

[275] H. L. Wang , H. F. Ma , M. Chen , S. Sun , T. J. Cui , Adv. Funct. Mater. 2021, 2100275.

[276] L. Bao , Q. Ma , R. Y. Wu , X. Fu , J. Wu , T. J. Cui , Adv. Sci. 2021, 8, 2100149. 10.1002/advs.202100149 PMC8336522 34038615

[277] S.‐J. Kim , I. Kim , S. Choi , H. Yoon , C. Kim , Y. Lee , C. Choi , J. Son , Y. W. Lee , J. Rho , Nanoscale Horiz. 2020, 5, 1088. 32377648 10.1039/d0nh00139b

[278] L. W. Wu , H. F. Ma , R. Y. Wu , Q. Xiao , Y. Gou , M. Wang , Z. X. Wang , L. Bao , H. L. Wang , Y. M. Qing , Adv. Opt. Mater. 2020, 8, 2001065.

[279] M. Wang , S. Xu , F. Yang , M. Li , IEEE Trans. Antennas Propag. 2019, 67, 6205.

[280] R. Y. Wu , L. Zhang , L. Bao , L. W. Wu , Q. Ma , G. D. Bai , H. T. Wu , T. J. Cui , Adv. Opt. Mater. 2019, 7, 1801429.

[281] W. H. Hayt Jr , J. E. Kemmerly , S. M. Durbin , Engineering Circuit Analysis (Eigth Edition), McGraw‐Hill Higher Education, New York 2006.

[282] C. A. Balanis , in Antenna Theory Analysis and Design, 3rd ed., Wiley, New York 2005, pp. 811–875.

[283] D. M. Pozar , Microwave Engineering, Wiley, New York 2011.

[284] J. Y. Dai , W. K. Tang , J. Zhao , X. Li , Q. Cheng , J. C. Ke , M. Z. Chen , S. Jin , T. J. Cui , Adv. Mater. Technol. 2019, 4, 1900044.

[285] L. Zhang , Z. X. Wang , R. W. Shao , J. L. Shen , X. Q. Chen , X. Wan , Q. Cheng , T. J. Cui , IEEE Trans. Antennas Propag. 2019, 68, 2984.

[286] S. Taravati , G. V. Eleftheriades , Phys. Rev. Appl. 2019, 12, 024026.

[287] S. Lin , S. Silva , J. Zhou , D. Talbayev , Adv. Opt. Mater. 2018, 6, 1800572.

[288] Y. Shi , Z. Yu , S. Fan , Nat. Photonics 2015, 9, 388.

[289] Y. Hadad , D. L. Sounas , A. Alu , Phys. Rev. B 2015, 92, 100304.

[290] Z. Wu , C. Scarborough , A. Grbic , Phys. Rev. Appl. 2020, 14, 064060.

[291] X. Guo , Y. Ding , Y. Duan , X. Ni , Light Sci. Appl. 2019, 8, 123. 31871675 10.1038/s41377-019-0225-z PMC6920367

[292] L. Zhang , X. Q. Chen , S. Liu , Q. Zhang , J. Zhao , J. Y. Dai , G. D. Bai , X. Wan , Q. Cheng , G. Castaldi , Nat. Commun. 2018, 9, 4334. 30337522 10.1038/s41467-018-06802-0 PMC6194064

[293] L. Zhang , X. Q. Chen , R. W. Shao , J. Y. Dai , Q. Cheng , G. Castaldi , V. Galdi , T. J. Cui , Adv. Mater. 2019, 31, 1904069. 10.1002/adma.201904069 31420926

[294] G. Castaldi , L. Zhang , M. Moccia , A. Y. Hathaway , W. X. Tang , T. J. Cui , V. Galdi , Adv. Funct. Mater. 2021, 31, 2007620.

[295] I. Alamzadeh , G. C. Alexandropoulos , N. Shlezinger , M. F. Imani , Sci. Rep. 2021, 11, 20737. 34671069 10.1038/s41598-021-99722-x PMC8528918

[296] G. C. Alexandropoulos , N. Shlezinger , P. Del Hougne , IEEE Commun. Mag. 2021, 59, 28.

[297] H. Gao , K. Cui , C. Huang , C. Yuen , IEEE Wireless Commun. Lett. 2021, 10, 2619.

[298] A. Zappone , M. Di Renzo , X. Xi , M. Debbah , IEEE Wireless Commun. Lett. 2020, 10, 464.

[299] Q. Tao , J. Wang , C. Zhong , IEEE Commun. Lett. 2020, 24, 2464.

[300] D. Li , IEEE Trans. Commun. 2021, 70, 1320.

[301] X. Pei , H. Yin , L. Tan , L. Cao , Z. Li , K. Wang , K. Zhang , E. Björnson , IEEE Trans. Commun. 2021, 69, 8627.

[302] J. Y. Dai , W. Tang , L. X. Yang , X. Li , M. Z. Chen , J. C. Ke , Q. Cheng , S. Jin , T. J. Cui , IEEE Trans. Antennas Propag. 2019, 68, 1618.

[303] H. Wymeersch , J. He , B. Denis , A. Clemente , M. Juntti , IEEE Veh. Technol. Mag. 2020, 15, 52.

[304] H. Zhang , H. Zhang , B. Di , K. Bian , Z. Han , L. Song , IEEE Trans. Wireless Commun. 2021, 20, 7743.

[305] G. C. Alexandropoulos , I. Vinieratou , H. Wymeersch , IEEE Wireless Commun. Lett. 2022, 11, 1072.

[306] H. Zhang , J. Hu , H. Zhang , B. Di , K. Bian , Z. Han , L. Song , IEEE Trans. Mob. Comput. 2020, 21, 2895.

[307] Y. U. Ozcan , O. Ozdemir , G. K. Kurt , IEEE Trans. Veh. Technol. 2021, 70, 2508.

[308] B. Paden , M. Čáp , S. Z. Yong , D. Yershov , E. Frazzoli , IEEE Trans. Intell. Veh. 2016, 1, 33.

[309] S. Chen , J. Hu , Y. Shi , Y. Peng , J. Fang , R. Zhao , L. Zhao , IEEE Commun. Stand. Mag. 2017, 1, 70.

[310] S. Fang , G. Chen , Y. Li , IEEE Wireless Commun. Lett. 2020, 10, 276.

[311] T. Shafique , H. Tabassum , E. Hossain , IEEE Trans. Commun. 2020, 69, 309.

[312] K. Keykhosravi , M. F. Keskin , G. Seco‐Granados , H. Wymeersch , in ICC 2021‐IEEE Int. Conf. on Communications, IEEE, Piscataway, NJ 2021, pp. 1–6.

[313] A. Elzanaty , A. Guerra , F. Guidi , M.‐S. Alouini , IEEE Trans. Signal Process. 2021, 69, 5386.

[314] E. Basar , I. Yildirim , F. Kilinc , IEEE Trans. Commun. 2021, 69, 8600.

[315] H.‐B. Jeon , S.‐H. Park , J. Park , K. Huang , C.‐B. Chae , IEEE Trans. Wirel. Commun. 2022, 21, 6478.

[316] L. Yang , F. Meng , J. Zhang , M. O. Hasna , M. Di Renzo , IEEE Trans. Veh. Technol. 2020, 69, 10385.

[317] X. Mu , Y. Liu , L. Guo , J. Lin , R. Schober , IEEE Trans. Wireless Commun. 2021, 20, 6648.

[318] X. Xie , F. Fang , Z. Ding , IEEE Trans. Veh. Technol. 2021, 70, 7705.

[319] F. Fang , Y. Xu , Q.‐V. Pham , Z. Ding , IEEE Trans. Veh. Technol. 2020, 69, 14088.

[320] J. Zuo , Y. Liu , Z. Qin , N. Al‐Dhahir , IEEE Trans. Commun. 2020, 68, 7170.

[321] Y. Zou , S. Gong , J. Xu , W. Cheng , D. T. Hoang , D. Niyato , IEEE Trans. Veh. Technol. 2020, 69, 12369.

[322] B. Lyu , P. Ramezani , D. T. Hoang , S. Gong , Z. Yang , A. Jamalipour , IEEE Trans. Commun. 2020, 69, 619.

[323] C. Pan , H. Ren , K. Wang , M. Elkashlan , A. Nallanathan , J. Wang , L. Hanzo , IEEE J. Sel. Areas Commun. 2020, 38, 1719.

[324] S. Dash , C. Psomas , I. Krikidis , I. F. Akyildiz , A. Pitsillides , IEEE Trans. Antennas Propag. 2022, 10.1109/TAP.2022.3142272

[325] Z. Wan , Z. Gao , F. Gao , M. Di Renzo , M.‐S. Alouini , IEEE Trans. Commun. 2021, 69, 4732.

[326] S. Elhoushy , M. Ibrahim , W. Hamouda , IEEE Wireless Commun. Lett. 2021, 11, 443.

[327] Y. Zhang , B. Di , H. Zhang , J. Lin , Y. Li , L. Song , IEEE Wireless Commun. Lett. 2020, 10, 775.

[328] Y. Zhang , B. Di , H. Zhang , J. Lin , C. Xu , D. Zhang , Y. Li , L. Song , IEEE Trans. Cogn. Commun. 2021, 7, 412.

[329] Y. Liu , Z. Qin , M. Elkashlan , Z. Ding , A. Nallanathan , L. Hanzo , Proc. IEEE 2017.

[330] W. Ni , X. Liu , Y. Liu , H. Tian , Y. Chen , IEEE Trans. Wirel. Commun. 2021, 20, 4253.

[331] Z. Yang , Y. Liu , Y. Chen , N. Al‐Dhahir , IEEE Trans. Commun. 2021, 69, 7414.

[332] Z. Zhang , C. Zhang , C. Jiang , F. Jia , J. Ge , F. Gong , IEEE Trans. Veh. Technol. 2021, 70, 4451.

[333] H. Q. Ngo , A. Ashikhmin , H. Yang , E. G. Larsson , T. L. Marzetta , IEEE Trans. Wireless Commun. 2017, 16, 1834.

[334] S. Elhoushy , M. Ibrahim , W. Hamouda , IEEE Commun. Surv. Tut. 2021, 24, 492.

[335] M. Bashar , K. Cumanan , A. G. Burr , H. Q. Ngo , H. V. Poor , IEEE Commun. Lett. 2018, 22, 1494.

[336] S. Huang , Y. Ye , M. Xiao , H. V. Poor , M. Skoglund , IEEE Wireless Commun. Lett. 2020, 10, 673.

[337] H. Yang , Z. Xiong , J. Zhao , D. Niyato , L. Xiao , Q. Wu , IEEE Trans. Wireless Commun. 2020, 20, 375.

[338] K. Feng , Q. Wang , X. Li , C.‐K. Wen , IEEE Wireless Commun. Lett. 2020, 9, 745.

[339] A. Al‐Hilo , M. Shokry , M. Elhattab , C. Assi , S. Sharafeddine , IEEE Trans. Veh. Technol. 2022, 71, 2333.

[340] J. Gao , X. Yi , C. Zhong , X. Chen , Z. Zhang , IEEE Wireless Commun. Lett. 2019, 8, 1727.

[341] P. Dong , H. Zhang , G. Y. Li , I. S. Gaspar , N. NaderiAlizadeh , IEEE J. Sel. Top. Signal Process. 2019, 13, 989.

[342] C. Huang , R. Mo , C. Yuen , IEEE J. Sel. Areas Commun. 2020, 38, 1839.

[343] C. Liaskos , A. Tsioliaridou , S. Nie , A. Pitsillides , S. Ioannidis , I. Akyildiz , in 2019 IEEE 20th International Workshop on Signal Processing Advances in Wireless Communications (SPAWC), IEEE, Piscataway, NJ 2019, pp. 1–5.

[344] J. Gao , C. Zhong , X. Chen , H. Lin , Z. Zhang , IEEE Commun. Lett. 2020, 24, 1052.

[345] A. Zappone , M. Di Renzo , M. Debbah , T. T. Lam , X. Qian , IEEE Veh. Technol. Mag. 2019, 14, 60.

[346] Z. Qin , H. Ye , G. Y. Li , B.‐H. F. Juang , IEEE Wireless Commun. 2019, 26, 93.

[347] A. Zappone , M. Di Renzo , M. Debbah , IEEE Trans. Commun. 2019, 67, 7331.

[348] A. M. Elbir , A. Papazafeiropoulos , P. Kourtessis , S. Chatzinotas , IEEE Wireless Commun. Lett. 2020, 9, 1447.

[349] L. Jiao , G. Sun , J. Le , K. Zeng , in Proc. of the 3rd ACM Workshop on Wireless Security and Machine Learning, ACM Press, New York 2021, pp. 61–66.

[350] K. K. Nguyen , S. Khosravirad , D. B. Da Costa , L. D. Nguyen , T. Q. Duong , IEEE J. Sel. Top. Signal Process. 2021, 16, 358.

[351] J. Zhao , L. Yu , K. Cai , Y. Zhu , Z. Han , IEEE J. Sel. Areas Commun. 2022, 40, 1287.

[352] M. Xu , S. Zhang , C. Zhong , J. Ma , O. A. Dobre , IEEE Commun. Lett. 2021, 25, 1921.

[353] B. Yang , X. Cao , C. Huang , C. Yuen , L. Qian , M. Di Renzo , IEEE Trans. Veh. Technol. 2021, 70, 3920.

[354] S. Liu , Z. Gao , J. Zhang , M. Di Renzo , M.‐S. Alouini , IEEE Trans. Veh. Technol. 2020, 69, 9223.

[355] S. Zhang , S. Zhang , F. Gao , J. Ma , O. A. Dobre , IEEE Trans. Commun. 2021, 69, 6691.

[356] H. Song , M. Zhang , J. Gao , C. Zhong , IEEE Commun. Lett. 2020, 25, 892.

[357] A. Mehmood , O. Waqar , M. M. U. Rahman , Phys. Commun. 2022, 51, 101558.

[358] A. M. Elbir , S. Coleri , IEEE Trans. Wirel. Commun. 2021, 21, 4255.

[359] W. Ni , Y. Liu , Z. Yang , H. Tian , X. Shen , IEEE Internet Things J. 2021, 9, 9608.

[360] L. Li , D. Ma , H. Ren , P. Wang , W. Lin , Z. Han , IEEE Trans. Green Commun. Network 2021, 6, 755.

[361] C. Huang , G. C. Alexandropoulos , C. Yuen , M. Debbah , in 2019 IEEE 20th Int. Workshop on Signal Processing Advances in Wireless Communications (SPAWC), IEEE, Piscataway, NJ 2019, pp. 1–5.

[362] J. Wang , C. Jiang , H. Zhang , Y. Ren , K.‐C. Chen , L. Hanzo , IEEE Commun. Surv. Tut. 2020, 22, 1472.

[363] A. Assra , J. Yang , B. Champagne , IEEE Trans. Veh. Technol. 2015, 65, 1229.

[364] T. Lin , Y. Zhu , IEEE Wireless Commun. Lett. 2019, 9, 103.

[365] W. Lee , M. Kim , D.‐H. Cho , IEEE Wireless Commun. Lett. 2018, 8, 141.

[366] Z. Wang , J. Qiu , Y. Zhou , Y. Shi , L. Fu , W. Chen , K. B. Letaief , IEEE Trans. Wireless Commun. 2021, 21, 808.

[367] T. Zhang , S. Mao , IEEE Trans. Green Commun. Network 2021.

[368] L. Li , D. Ma , H. Ren , D. Wang , X. Tang , W. Liang , T. Bai , China Commun. 2020, 17, 115.

[369] D. Kitayama , Y. Hama , K. Goto , K. Miyachi , T. Motegi , O. Kagaya , Opt. Express 2021, 29, 29292. 34615041 10.1364/OE.435648

[370] H. Zhang , S. Zeng , B. Di , Y. Tan , M. Di Renzo , M. Debbah , Z. Han , H. V. Poor , L. Song , IEEE Commun. Mag. 2022, 60, 39.

[371] S. Zhang , H. Zhang , B. Di , Y. Tan , Z. Han , L. Song , IEEE Trans. Veh. Technol. 2020, 69, 13905.

[372] C. Wu , Y. Liu , X. Mu , X. Gu , O. A. Dobre , IEEE Commun. Lett. 2021, 25, 3036.

[373] C. De Lima , D. Belot , R. Berkvens , A. Bourdoux , D. Dardari , M. Guillaud , M. Isomursu , E.‐S. Lohan , Y. Miao , A. N. Barreto , IEEE Access 2021, 9, 26902.

[374] H. Niu , Z. Chu , F. Zhou , P. Xiao , N. Al‐Dhahir , IEEE Trans. Veh. Technol. 2022, 71, 2122.

[375] H. Niu , X. Liang , IEEE Syst. J. 2022, 10.1109/JSYST.2022.3159551.

[376] C. Wu , C. You , Y. Liu , X. Gu , Y. Cai , IEEE Commun. Lett. 2021, 26, 652.

[377] C. Zhang , W. Yi , Y. Liu , Z. Ding , L. Song , IEEE Trans. Wireless Commun. 2022, 21, 7753.

[378] C. Wu , Y. Liu , X. Mu , X. Gu , O. A. Dobre , IEEE Commun. Lett. 2021, 25, 3036.

[379] C. Wu , X. Mu , Y. Liu , X. Gu , X. Wang , IEEE Trans. Wireless Commun. 2022, 21, 6861.

[380] Y. Han , N. Li , Y. Liu , T. Zhang , X. Tao , IEEE Wireless Commun. Lett. 2022, 11, 1191.

[381] S. Zhang , H. Zhang , B. Di , Y. Tan , M. Di Renzo , Z. Han , H. V. Poor , L. Song , IEEE Trans. Wireless Commun. 2021, 21, 219.

[382] J. Zuo , Y. Liu , Z. Ding , L. Song , in 2021 IEEE Global Communications Conf. (GLOBECOM), IEEE, Piscataway, NJ 2021, pp. 1–6.

[383] C. Zhang , W. Yi , K. Han , Y. Liu , Z. Ding , M. Di Renzo , in 2021 IEEE Global Communications Conf. (GLOBECOM), IEEE, Piscataway, NJ 2021, pp. 1–6.

[384] T. Hou , J. Wang , Y. Liu , X. Sun , A. Li , B. Ai , IEEE Trans. Veh. Technol. 2021, 71, 1043.

[385] S. Fang , G. Chen , Z. Abdullah , Y. Li , IEEE Commun. Lett. 2022, 26, 1231.

[386] M. Chen , H. V. Poor , W. Saad , S. Cui , IEEE Trans. Wireless Commun. 2020, 20, 2457.

[387] W. Ni , Y. Liu , Y. C. Eldar , Z. Yang , H. Tian , in 2021 IEEE Global Communications Conf. (GLOBECOM), IEEE, Piscataway, NJ 2021, pp. 1–6.

[388] E. Worrell , J. Allwood , T. Gutowski , Annu. Rev. Environ. Resour. 2016, 41, 575.

[389] B. Mao , Y. Kawamoto , J. Liu , N. Kato , IEEE Commun. Lett. 2019, 23, 2130.

[390] G. Vallero , D. Renga , M. Meo , M. A. Marsan , IEEE Trans. Network Serv. Manag. 2019, 16, 896.

[391] B. Mao , F. Tang , Y. Kawamoto , N. Kato , IEEE Commun. Surv. Tut. 2021, 24, 210.

